# Overall reaction mechanism of photocatalytic CO_2_ reduction on a Re(i)-complex catalyst unit of a Ru(ii)–Re(i) supramolecular photocatalyst†

**DOI:** 10.1039/d3sc06059d

**Published:** 2023-12-21

**Authors:** Kei Kamogawa, Yuki Kato, Yusuke Tamaki, Takumi Noguchi, Koichi Nozaki, Tatsuo Nakagawa, Osamu Ishitani

**Affiliations:** a Department of Chemistry, School of Science, Tokyo Institute of Technology 2-12-1-NE-2 O-okayama, Meguro-ku Tokyo 152-8550 Japan ishitani@chem.titech.ac.jp; b Department of Physics, Graduate School of Science, Nagoya University Nagoya 464-8602 Japan yuki.kato@bio.phys.nagoya-u.ac.jp; c Department of Chemistry, Graduated School of Science and Engineering, University of Toyama 3190, Gofuku, Toya-ma-shi Toyama 930-8555 Japan; d UNISOKU Co., Ltd 2-4-3 Kasugano, Hirakata Osaka 573-0131 Japan; e Department of Chemistry, Graduate School of Advanced Science and Engineering, Hiroshima University 1-3-1 Kagamiyama, Higashi-Hiroshima Hiroshima 739 8526 Japan

## Abstract

Rhenium(i) complexes *fa*c-[Re^I^(diimine)(CO)_3_(L)]^*n*+^ are mostly used and evaluated as photocatalysts and catalysts in both photochemical and electrochemical systems for CO_2_ reduction. However, the selective reduction mechanism of CO_2_ to CO is unclear, although numerous mechanistic studies have been reported. A Ru(ii)–Re(i) supramolecular photocatalyst with *fac*-[Re^I^(diimine)(CO)_3_{OC(O)OCH_2_CH_2_NR_2_}] (R = C_2_H_4_OH) as a catalyst unit (RuC2Re) exhibits very high efficiency, selectivity, and durability of CO formation in photocatalytic CO_2_ reduction reactions. In this work, the reaction mechanism of photocatalytic CO_2_ reduction using RuC2Re is fully clarified. Time-resolved IR (TR-IR) measurements using rapid-scan FT-IR spectroscopy with laser flash photolysis verify the formation of RuC2Re(COOH) with a carboxylic acid unit, *i.e.*, *fac*-[Re^I^(diimine)(CO)_3_(COOH)], in the photocatalytic reaction solution. Additionally, this important intermediate is detected in an actual photocatalytic reaction using steady state irradiation. Kinetics analysis of the TR-IR spectra and DFT calculations demonstrated the reaction mechanism of the conversion of the one-electron reduced species of RuC2Re with a *fac*-[Re^I^(diimine˙^−^)(CO)_3_{OC(O)OCH_2_CH_2_NR_2_}]^−^ unit, which was produced *via* the photochemical reduction of RuC2Re by 1,3-dimethyl-2-phenyl-2,3-dihydro-1*H*-benzo[*d*]imidazole (BIH), to RuC2Re(COOH). The kinetics of the recovery processes of the starting complex RuC2Re from RuC2Re(COOH) accompanying the release of CO and OH^−^ was also clarified. As a side reaction of RuC2Re(COOH), a long-lived carboxylate–ester complex with a *fac*-[Re^I^(diimine)(CO)_3_(COOC_2_H_4_NR_2_)] unit, which was produced by the nucleophilic attack of TEOA to one of the carbonyl ligands of RuC2Re(CO) with a *fac*-[Re^I^(diimine)(CO)_4_]^+^ unit, was formed during the photocatalytic reaction. This complex works not only as a precursor in another minor CO formation process but also as an external photosensitiser that photochemically reduces the other complexes *i.e.*, RuC2Re, RuC2Re(COOH), and the intermediate that is reductively converted to RuC2Re(COOH).

## Abstract 2

The whole mechanism of photocatalytic CO_2_ reduction to CO by a Ru(ii)–Re(i) supramolecular photocatalyst was successfully clarified using time-resolved infrared spectroscopy, UHPLC analysis, electrochemical measurements, and DFT calculations.

## Graphical abstract

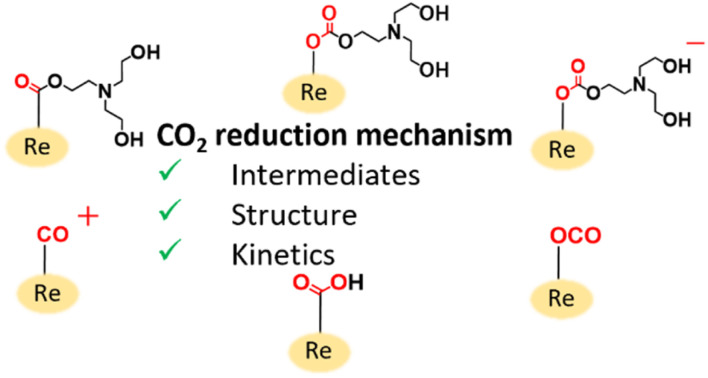

## Introduction

Photocatalytic CO_2_ reduction using visible light as an energy source has the potential to solve three serious problems for humankind, *i.e.*, global warming and the shortage of both energy and carbon resources.^1,2^ In particular, the two-electron reduction from CO_2_ to CO, that can proceed at relatively low overpotentials *via* proton coupled two-electron reduction (eqn (1) at pH 7) or reductive disproportionation (eqn (2)), has attracted attention because CO is a useful intermediate for the synthesis of high-energy and useful carbon materials.

1 CO_2_ + 2H^+^ + 2e^−^ → CO + H_2_O, *E*° = −0.53 V *vs.* NHE

2 2CO_2_ + 2e^−^ → CO + CO_3_^2−^, *E*° = −0.64 V *vs.* NHE

In 1983, *fac*-[Re(bpy)(CO)_3_X] (bpy = 2,2′-bipyridine, X = Cl or Br) was first reported by Lehn *et al.* as an efficient CO_2_ reduction photocatalyst. This photocatalytic reaction proceeded in a mixed solution of *N*,*N*′-dimethylformamide (DMF) and triethanolamine (TEOA) (5 : 1 v/v) with the very high selectivity of CO as a reduction product without the formation of H_2_ even in the presence of water and without the formation of formic acid. After this report, many transition-metal-complex catalysts such as Re(i),^3–7^ Ru(ii),^8,9^ Fe(ii),^10–13^ Co(i),^10,14–18^ Ni(i)^19–21^ and Mn(i)^22,23^ complexes were reported for the photocatalytic reduction of CO_2_ to CO. Although mechanistic studies on these photocatalytic reactions have been continuously reported, mechanistic insights into the photocatalytic reactions, such as the structures of the intermediates and reaction rates of each process during the photocatalytic CO_2_ reduction, have been insufficient for the systematic design of better photocatalysts and the addition of new functions to the photocatalysts.

A typical example is the mechanism of photocatalytic CO_2_ reduction using *fac*-[Re(diimine)(CO)_3_X] as the photocatalyst. Many researchers have been interested in the selective formation mechanism of CO in photocatalytic CO_2_ reduction as well as the electrocatalytic reduction of CO_2_ using the Re complexes as catalysts.^3–7,24^ Kubiak *et al.* used the stopped-flow method with a rapid scan FT-IR detector in the reaction of a five-coordinated 18e^−^ species [Re(4,4′-di-*tert*-butyl-bpy)(CO)_3_]^−^ with CO_2_ and detected a carboxylic acid complex.^25^ In many systems using metal-complex photocatalysts that reduce CO_2_ to CO, the corresponding carboxylic acid complexes have been assumed to be intermediates based on the mechanistic studies on chemical and/or electrocatalytic CO_2_ reduction.^1,25–40^ However, the mechanism of the photocatalytic reaction was too potentially different to be clarified based on only the mechanisms of the chemical and electrochemical CO_2_ reduction reactions even when using the same catalyst; this is because the initial step of the photocatalytic reactions was a one-electron reduction of the catalyst but not a two-electron reduction. In other words, the one-electron reduced catalyst has an adequate lifetime for changing its structure, *i.e.*, the formation of another intermediate or other intermediates before accepting the second electron from another molecule with sufficient reduction power to reduce the intermediate(s) that proceeds *via* diffusion collision. This second electron injection process to the intermediate(s) made from the one-electron reduced catalysts is considerably slower compared to those in the electrocatalytic CO_2_ reduction reactions in which the electrode can simultaneously supply electrons.^1^

In the reaction mechanism of the “photocatalytic” CO_2_ reduction using the Re catalysts, Inoue *et al.* identified an “oxidized” carboxylic acid complex [Re^II^(dmb)(CO)_3_(COOH)]^+^ (dmb = 4,4′-dimethyl-2,2′-bipyridyl) in the photocatalytic reaction of *fac*-[Re(dmb)(CO)_3_Cl] using triethylamine (TEA) as a sacrificial electron donor in a DMF solution using cold-spray ionization spectroscopy and operando measurements using XAFS and FT-IR.^27,28^ Fujita *et al.* identified a di-nuclear Re complex with a carboxylate bridging ligand when the penta-coordinated 17e^−^ species [Re(dmb)(CO)_3_]^0^ was produced *via* the photocleavage of the Re–Re bond of [Re(dmb)(CO)_3_]_2_ in CO_2_ saturated dry DMF.^41,42^

Notably, in most reported efficient photocatalytic reactions using the Re-complex catalyst and/or photocatalyst including Lehn's system, DMF or *N*,*N*′-dimethylacetamide (DMA) containing a high concentration of TEOA, typically DMF : TEOA = 5 : 1, the use of these solutions can enhance the durability, efficiency, and selectivity of the photocatalytic CO_2_ reduction to CO compared with those using other solvents and additives such as MeCN and TEA.^3–6,43^ We previously demonstrated that one of the advantages of the high concentration of TEOA is that TEOA assists CO_2_ capture into the Re(i) complex (eqn (3)).^5^ In the photocatalytic reaction by *fac*-[Re(bpy)(CO)_3_Br] in the DMF–TEOA (5 : 1 v/v) mixed solution, *i.e.*, same as Lehn's system, it was elucidated that in the initial stage of the photocatalytic reaction, *fac*-[Re(bpy)(CO)_3_Br] is rapidly converted to the corresponding carbonate ester complex, *i.e.*, *fac*-[Re(bpy)(CO)_3_{OC(O)OCH_2_CH_2_NR_2_}] (R = C_2_H_4_OH) that acts as a catalyst for CO_2_ reduction, and the residual *fac*-Re(bpy)(CO)_3_Br acts as a redox photosensitiser that initiates photochemical one-electron transfer from TEOA to *fac*-[Re(bpy)(CO)_3_{OC(O)OCH_2_CH_2_NR_2_}]. In many photocatalytic systems using Re(i) complexes as a “photocatalyst” in the presence of TEOA, similar reactions are expected, *i.e.*, these photocatalytic systems work as two-component systems including the carbonate ester Re(i) complex as a catalyst and the starting emissive Re(i) complex, with an excited state that can be reductively quenched by TEOA, as a photosensitiser. The addition of [Ru(diimine)_3_]^2+^ as an additional photosensitiser and a suitable reductant drastically enhances the photocatalysis of the system including the Re(i) complex and TEOA.^44,45^3
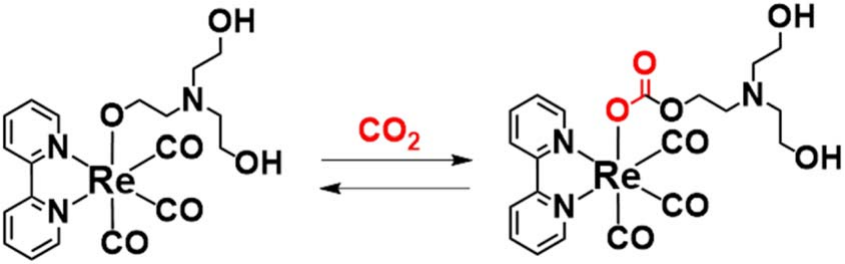


Supramolecular photocatalysts, in which the [Ru(diimine)_3_]^2+^ redox photosensitiser and Re(i) catalyst units are connected by an ethylene chain, promote higher photocatalytic activity compared with a mixed system involving corresponding mononuclear Ru(ii) and Re(i) complexes owing to rapid intramolecular electron transfer from the photosensitiser to the catalyst unit.^45^ From a mechanistic viewpoint, supramolecular photocatalysts are useful because the rapid electron transfer process from the reduced photosensitiser to the catalyst can be kinetically separated from CO_2_ reduction processes on the reduced Re unit, which are much slower compared to the intramolecular electron transfer. In these systems, we can omit the direct photoexcitation of the Re complexes using long-wavelength light (typically 480 nm), which only the Ru photosensitiser unit can absorb.

**Scheme 1:**
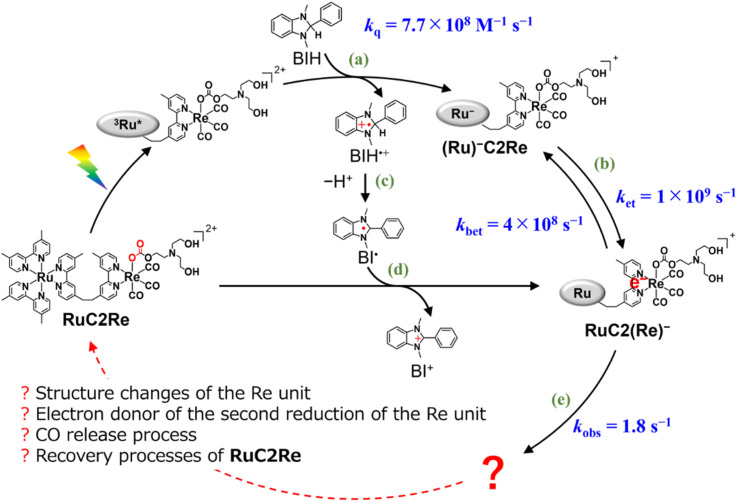
Formation processes of the one-electron reduced species (OERS) of RuC2Re and unknown processes shown as “
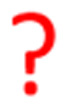
”.

In the photocatalytic system using a supramolecular photocatalyst consisting of the Ru(ii) photosensitiser and *fac*-[Re(diimine)(CO)_3_{OC(O)OC_2_H_4_NR_2_}] catalyst units (RuC2Re), kinetic studies on the formation processes of the one-electron reduced species (OERS) of the Re unit, which is one of the key intermediates in the initial stage of photocatalytic CO_2_ reduction, and its reactivity, were clarified using time-resolved IR (TR-IR) measurements using the pump–probe method and TR-vis measurements using the Randomly Interleaved Pulse Train (RIPT) method (Scheme 1).^46,47^ These results clearly indicated that the OERS of RuC2Re is produced by two processes: (1) a fast process lasting several tens of nanoseconds after excitation, which is reductive quenching of the excited state of the Ru unit to yield the OERS of the Ru unit, *i.e.*, (Ru)^−^C2Re (Scheme 1, Process (a)), followed by fast intramolecular electron transfer to the Re unit to produce another OERS, *i.e.*, RuC2(Re)^−^ in which the Re unit accepts one electron (Process (b)); and (2) a slower process lasting several tens of microseconds, which is attributed to the reduction of RuC2Re in the ground state by BI˙ (Process (d)), which is produced by the deprotonation of BIH˙^+^ (Process (c)).

The intramolecular electron transfer from the reduced Ru unit to the Re unit, with a rate constant (*k*_et_ = 1 × 10^9^ s^−1^, *k*_bet_ = 4 × 10^8^ s^−1^) that was determined in this study, was a much faster process compared to the following processes; the two intermediates (Ru)^−^C2Re and RuC2(Re)^−^ could be spectroscopically observed. After the intramolecular electron transfer, the subsequent reaction of the RuC2(Re)^−^ (Process (e)) proceeded at a relatively slow rate (*k*_obs_ = 1.8 ± 0.1 s^−1^ at 298 K). The intermediates produced after this slow reaction could not be observed because it was difficult to prevent the diffusion of transient species in the solution during the measurements using the pump–probe and RIPT methods.^48–51^

In this work, we successfully clarified the unknown subsequent reaction mechanism after the intramolecular electron transfer in the photocatalytic CO_2_ reduction on the Re catalyst unit, which is shown as “?” in Scheme 1, by applying various methods, *i.e.*, TR-IR spectroscopy using the rapid scan FT-IR method, which is more suitable for tracking the reaction that proceeds on the time scale from milliseconds to minutes, liquid chromatography analysis of the photocatalytic reaction solutions, and DFT calculations. This is the first report on the whole picture of the CO_2_ reduction mechanism on the Re catalyst in the photocatalytic systems.

## Results

### Rapid-scan time-resolved FT-IR measurements coupled with laser flash photolysis

A CO_2_ saturated DMSO–TEOA (5 : 1 v/v) mixed solution containing RuC2Re (1.0 mM) and BIH (0.1 M) as a sacrificial electron donor in an optical cell was irradiated with the second harmonic of a pulsed Nd : YAG laser (532 nm) once every 10 min. Notably, under these reaction conditions using an LED continuous light source at *λ*_max_ = 530 nm instead of laser pulses, RuC2Re can reduce CO_2_ to CO with high TON (>2000), quantum yield (=40%), and selectivity (>99%), which is similar to the photocatalysis of RuC2Re measured in the DMA–TEOA (5 : 1 v/v) mixed solution.^46,52^ Because the photocatalyst was finally recovered to the original structure within the pulse interval (10 min) described below, the solution did not flow during the measurements. In addition, because of the large diameter of the excitation pulse (∼1 cm), the diffusion of the transient species was not a problem for the measurements. RuC2Re shows vibrational absorption bands of the CO ligands at 2018, 1911, and 1888 cm^−1^, the C=O moiety of the carbonate ester ligand at 1668 cm^−1^ and both the dmb ligands of the Ru and Re units at 1619 cm^−1^ (Fig. S1†).^46^

**Fig. 1:**
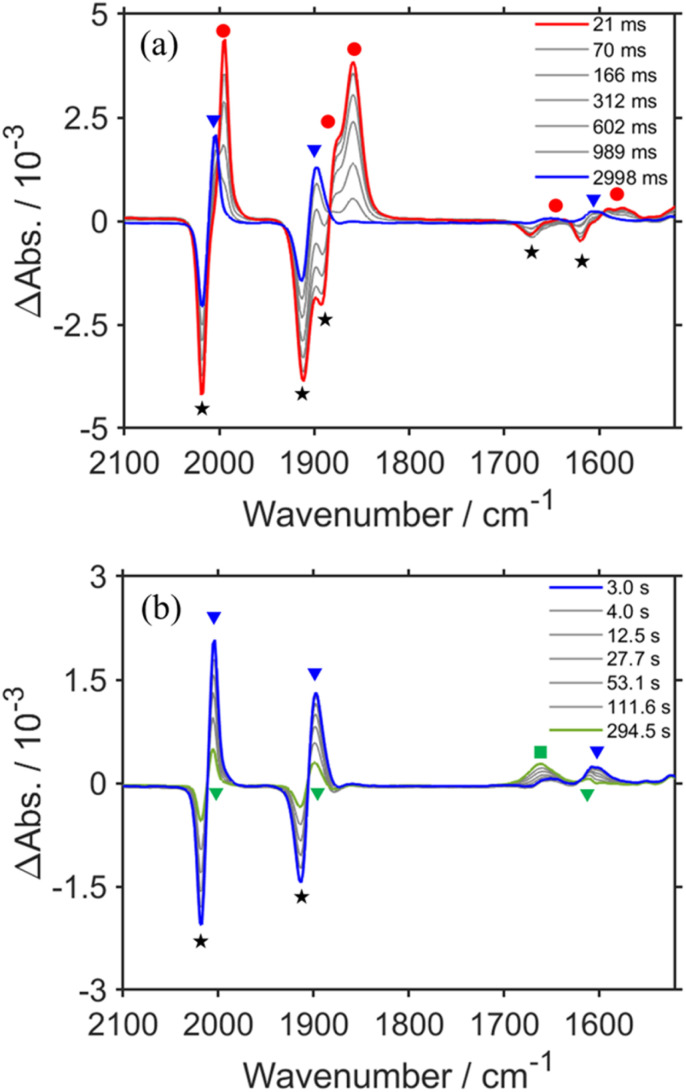
TR-IR spectra of CO_2_ saturated DMSO–TEOA (5 : 1 v/v) solution containing RuC2Re (1.0 mM) and BIH (0.1 M) (a) from 21 ms to 3.0 s and (b) from 3.0 s to 5.0 min after pulsed excitation at 532 nm. A total of 50 loops of spectra using the two samples were averaged for the final data.

Fig. 1a shows the TR-IR spectra from 21 ms to 3.0 s after the laser flash illumination. At 21 ms, there was an appearance of bleaching bands at 2019, 1911, 1894 (sh), 1672, and 1620 cm^−1^ (marked with black stars in Fig. 1a), which originated from the consumption of RuC2Re, and new absorption bands at 1994, 1876 (sh), 1859, 1639, 1590 and 1575 cm^−1^ (marked with red circles), which were attributed to the OERS of the Re unit RuC2(Re)^−^.^46,53–55^ These spectral changes are identical to the previously reported TR-IR spectra measured by the pump–probe method at several ns to ms after laser irradiation.^46^ These results clearly indicate that we could successfully apply the rapid-scan time-resolved FT-IR measurements with the laser flash photolysis to the photochemical formation of RuC2(Re)^−^, which is produced by electron transfer from BIH to the excited Ru unit, and intramolecular electron transfer from the reduced Ru unit to the Re unit as well as the reduction of RuC2Re by BI˙ as described in the Introduction section (Scheme 1).

DFT calculations (Table S1†) were performed to obtain the *ν*_CO_ values of a model mononuclear complex of the Re catalyst unit of RuC2Re and RuC2(Re)^−^, *i.e.*, *fac*-[Re(dmb)(CO)_3_{OC(O)OC_2_H_4_NR_2_}] (R = CH_2_CH_2_OH, dmb = 4,4′-dimethyl-2,2′-bipyridine) (Re) and its OERS (Re^−^). It should be noted that the electronic interactions between the Ru and Re units, which are connected by the ethylene chain in RuC2Re and its derivatives, are weak.^46,56,57^ The results are presented in Table S1,† in which the calculated and experimental *ν*_CO_ values are in good agreement. Therefore, we used the DFT calculation as one of the methods for investigating the short-lived intermediates as described below.

**Scheme 2:**
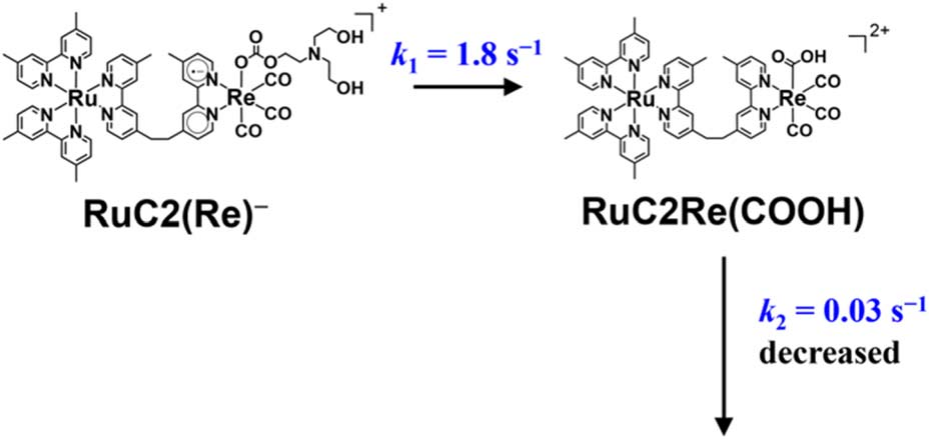
Conversion reaction from RuC2(Re)^−^ to RuC2Re(COOH) and the rate constant of the following process.

From 21 ms after the laser flash, the positive bands of RuC2(Re)^−^ slowly decayed and new peaks were clearly observed at 2004, 1898, and 1606 cm^−1^ (marked with blue triangles in Fig. 1a) with isosbestic points at 2001, 1884, and 1595 cm^−1^. These changes were completed until 3.0 s after the laser flash. During this period, the bleaching bands decayed to approximately half of their intensity. The newly appeared peaks were very similar to those of a mononuclear carboxylic acid complex *fac*-[Re^I^(dmb)(CO)_3_(COOH)] (Fig. S2†), which showed *ν*_CO_s at 2004 and 1893 cm^−1^; two other peaks were observed at 1620 and 1606 cm^−1^ as well. The product in this time scale is attributable to a carboxylic acid complex RuC2Re(COOH), of which the Re unit has a *fac*-[Re^I^(diimine)(CO)_3_(COOH)] structure (Scheme 2). Because the calculated vibrational energy of the stretching band of the C=O group in the carboxylic acid ligand of *fac*-[Re^I^(dmb)(CO)_3_(COOH)] was smaller than that of the dmb ligand (Table S1†), the peaks at 1620 and 1606 cm^−1^ in the IR of *fac*-[Re^I^(dmb)(CO)_3_(COOH)] were attributed to the stretching bands of the dmb ligand and the C=O group of the carboxylic acid ligand, respectively. The peak attributable to the dmb stretching vibration of the Re unit of RuC2Re(COOH) was not clearly identified in the TR-IR spectra likely because the energy of this vibration is very similar to those of both the Ru and Re units of RuC2Re. These peak assignments are summarized in Table 1.

**Table 1:** Vibrational bands observed in TR-IR measurements and their assignments

Complex	IR absorption/cm^−1^
RuC2Re (★)	2019, 1911, 1894 (sh) [CO ligands]
	1672 [C=O of the carbonate ester ligand]
	1620 [dmb ligand]
RuC2(Re)^−^ 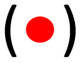	1994, 1876 (sh), 1859 [CO ligand]
	1639 [C=O of the carbonate ester ligand]
	1590, 1575 [dmb ligand]
RuC2Re(COOH) 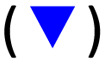	2004, 1898 [CO ligands]
	1606 [C=O of the carboxylic acid ligand]
RuC2Re(CO-TEOA) 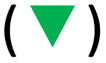	2006, 1900 [CO ligands]
	1611 [C=O of the carboxylate ester ligand]
HCO_3_^−^ 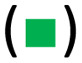	1662

**Fig. 2:**
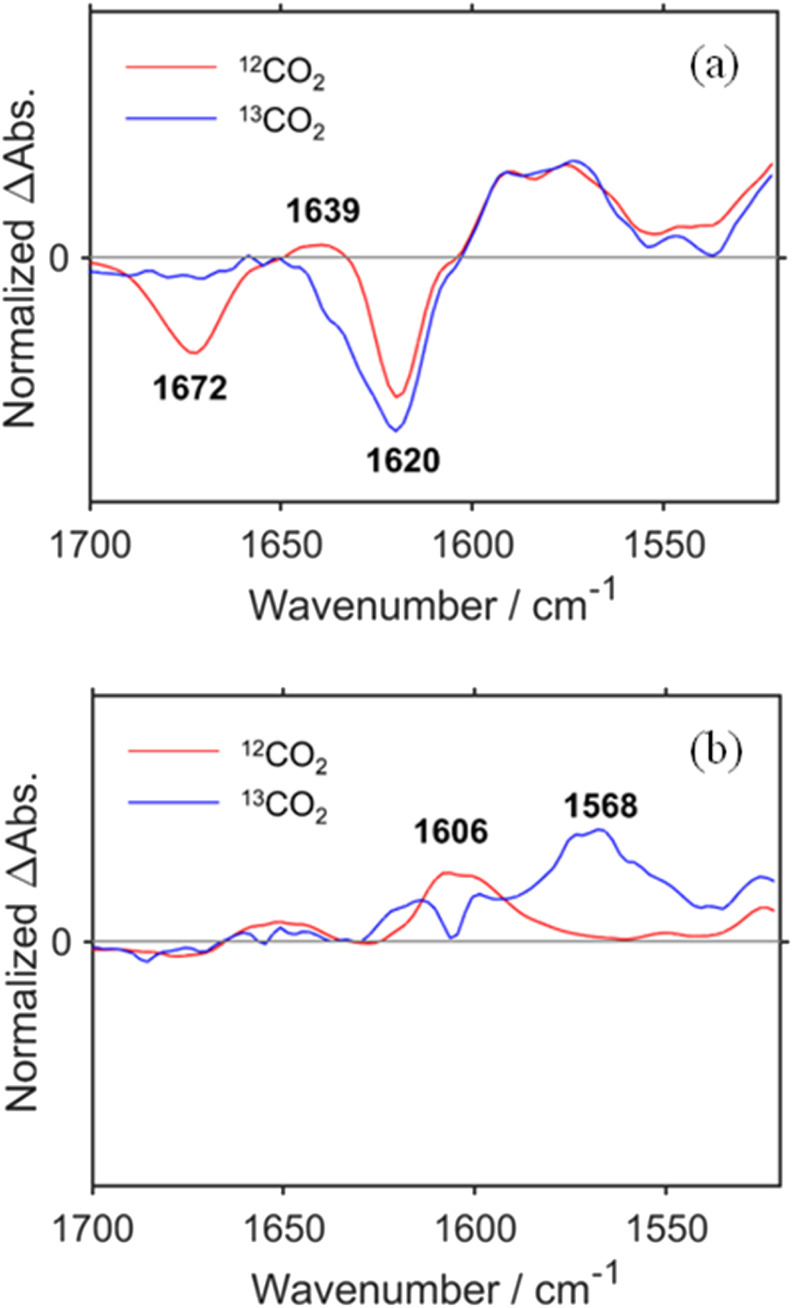
TR-IR spectra measured in ordinary CO_2_ (red) and ^13^CO_2_ (blue) saturated DMSO–TEOA (5 : 1 v/v) solution containing RuC2Re (1.0 mM) and BIH (0.1 M) at (a) 21 ms and (b) 3.0 s after the laser flash. These spectra were normalized by absorbance at 2019 cm^−1^ at 21 ms after pulsed excitation.

To confirm the peak assignments described above, the same experiments were performed under a ^13^CO_2_ atmosphere. The broad negative band at *ν*_max_ = 1672 cm^−1^, which was observed under an ordinary CO_2_ atmosphere, was not observed at the TR-IR spectrum measured immediately after the laser flash, and another negative peak was observed as a shoulder at ∼1640 cm^−1^ (Fig. 2a and S3a†). This strongly supports that these negative bands, *i.e.*, *ν*_max_ = 1672 cm^−1^ under ordinary CO_2_ and *ν*_shoulder_ ∼ 1640 cm^−1^ under ^13^CO_2_, were attributed to the C=O stretching of the carbonyl ester ligand of RuC2Re. The weak positive band at *ν*_max_ = 1639 cm^−1^ observed under ordinary CO_2_, which is attributed to the C=O vibrational band of RuC2(Re)^−^, was not observed under ^13^CO_2_ and a replacement peak was not clearly observed as well. This is reasonable because the band under ^13^CO_2_ was shifted to a lower frequency and was overlapped by the stronger negative peak of the dmb ligand at *ν*_max_ = 1620 cm^−1^, which was observed both under ordinary CO_2_ and under ^13^CO_2_. The bands attributed to the CO ligands (bleaching bands at 2019, 1911 and 1894 (sh) cm^−1^; positive bands at 1994, 1876 (sh), and 1859 cm^−1^) did not change. At 3.0 s after laser irradiation (Fig. 2b and S3b†), the positive peak attributable to the C=O vibrational band of the carboxyl ligand of RuC2Re(COOH) observed at 1606 cm^−1^ under ordinary CO_2_ was shifted to the 38 cm^−1^ lower wavenumber under ^13^CO_2_, *i.e.*, *ν*_max_ = 1568 cm^−1^ (although small shoulder peaks were also observed at 1992 and 1859 cm^−1^ as shown in Fig. S3b,† these should be attributed to the ^13^CO ligand(s) formed during the TR-IR measurements).^58^ This lower shift of the C=O vibration of the carboxylic acid ligand is consistent with previous reports on [Re^II^(dmb)(CO)_3_(COOH)]^+^ (−39 cm^−1^) and the iron porphyrin carboxylic acid complex (−48 cm^−1^).^28,36^ It was also supported by the vibrational frequency predictions using the DFT calculation (−37 cm^−1^; Table S1†). These results demonstrate that the carbonate ester ligand of RuC2Re originated from CO_2_ and was converted to the carboxylic acid ligand during the photocatalytic reaction. In addition, similar experiments using TEOA with deuterated hydroxy groups and deuterated BIH at the second position of the dihydroimidazole ring were conducted with ordinary CO_2_. The peak attributable to the C=O stretching of the carbonyl ester ligand of RuC2Re(COOH) shifted to the 13 cm^−1^ lower wavenumber (Fig. S4†). This lower shift also supports the identification of the C=O stretching band because the DFT calculation of the complex with the *fac*-[Re^I^(diimine)(CO)_3_(COOD)] structure indicates that deuteration induces a lower shift of the C=O stretching band by −9 cm^−1^ compared to that with *fac*-[Re^I^(diimine)(CO)_3_(COOH)].

The TR-IR spectra from 21 ms to 4.0 s after the laser flash were analyzed using a global sequential routine.^59,60^ A good fitting was achieved by analysis using double components (Fig. S5†). The obtained evolution associated spectra (EAS) indicated that the first and second components were assigned to RuC2(Re)^−^ and RuC2Re(COOH), respectively. The rate constants were determined to be *k*_1_ = 1.8 ± 0.1 s^−1^ and *k*_2_ = 0.031 ± 0.008 s^−1^ (Scheme 2); the experimental errors were calculated using data from three independent experiments. Because *k*_1_ is very close to the previously reported rate constant (1.8 ± 0.1 s^−1^) of the subsequent reaction of RuC2(Re)^−^, which was measured using UV-vis absorption spectroscopy,^46^ the rate constants are assigned to the conversion reaction from RuC2(Re)^−^ to RuC2Re(COOH) and the slower subsequent reaction of RuC2Re(COOH) (Scheme 2), which is discussed in details below. Notably, as previously reported, the faster spectral change could be fitted with a single exponential function, *i.e.*, its reaction rate linearly depends on the concentration of the OERS of RuC2Re.

Fig. 1b shows the TR-IR spectra from 3.0 s to 5 min after the laser flash. The positive peak assigned to RuC2Re(COOH) decayed. Although the recovery of the bleaching bands assigned to the starting complex RuC2Re was observed, it did not fully recover (approximately 90% recovery after 5 min) as described in detail below. In addition, a new peak was observed at 1662 cm^−1^ (marked with a green square in Fig. 1b), which is attributable to HCO_3_^−^ (Table 1): this identification was also supported by the fact that this peak shifted to the 44 cm^−1^ lower wavenumber in the same experiments under the ^13^CO_2_ atmosphere (Fig. S3c and d†), which is consistent with the reported shift of HCO_3_^−^.^61^ We reported that the amount of HCO_3_^−^ produced was similar to that of CO during the photocatalytic CO_2_ reduction reaction using RuC2Re and BIH, and the overall reaction equation of the photocatalytic reaction is presented in eqn (4).^46^

4 2CO_2_ + BIH → CO + HCO_3_^−^ + BI^+^

In the TR-IR measurements under the ^13^CO_2_ atmosphere, the bleaching and positive bands assigned to RuC2Re(COOH) decayed and, in addition, another peak appeared at 1862 cm^−1^, which is attributed to the ^13^CO ligand increased during 5 min after the irradiation (Fig. S3c†). This result indicates that the ^12^CO ligand attached on the central Re was sometimes released and the ^13^CO ligand can remain in the Re complex during the TR-IR measurements. It has been reported that, in other photocatalytic reactions using similar Re(i) complexes as catalysts under a ^13^CO_2_ atmosphere, the ^12^CO ligands were gradually substituted with ^13^CO.^27,58^

Because ∼90% of the OERS of RuC2Re recovered to the non-reduced RuC2Re at 5 min after the irradiation (Fig. 1b), most of the CO formation from RuC2Re(COOH) proceeded during this period of time in the TR-IR measurement. This suggests that the CO formation from RuC2Re(COOH) is the rate-determining step or one of the rate-determining steps in the TR-IR measurement. It was reported that the photocatalytic CO_2_ reduction proceeded with almost the same CO production rate (turnover frequency) when the photocatalytic reactions using a Ru(ii)–Re(i) supramolecular photocatalyst were conducted between under pure CO_2_ and under Ar containing 10% CO_2_ atmospheres, *i.e.*, the photocatalytic reaction rate does not depend on the CO_2_ concentration under these reaction conditions. These results suggest that RuC2Re(COOH) released CO and OH^−^, and the released OH^−^ reacted with another CO_2_ to form HCO_3_^−^.

**Fig. 3:**
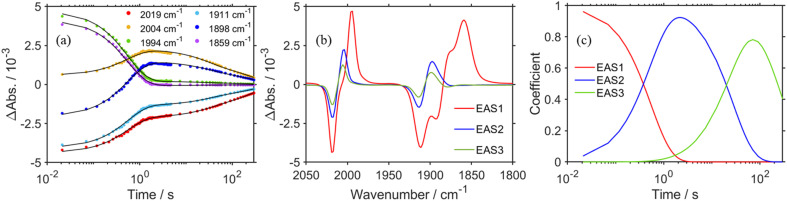
(a) Kinetics traces (dots) of TR-IR spectra from 21 ms to 5.0 min at characteristic wavelengths with their fits obtained by global analysis using a three component global sequential fitting routine (black line). (b) Evolution-associated spectra (EAS) generated by global analysis of TR-IR spectra from 21 ms to 5 min after laser flash using three components. (c) Time-courses of EAS.

The positive peaks of RuC2Re(COOH) observed at 3 s after the laser flash were upshifted slightly but clearly to 2006, 1900, and 1611 cm^−1^ without shifts of the bleaching band (Fig. 1b and S6†); these blue-shifted peaks remained even at 5 min after the laser flash as described above. The TR-IR spectral change between 2100 and 1700 cm^−1^ up to 5 min after the laser flash could be globally fitted with three components (Fig. 3): the low wavenumber region (1550–1700 cm^−1^) was excluded because of the effect of the accumulated HCO_3_^−^. The first (EAS1; *k*_1_ = 1.8 ± 0.1 s^−1^) and second (EAS2; 
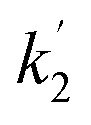
 = (4 ± 1) × 10^−2^ s^−1^) were similar to the results of the aforementioned global analysis of all the TR-IR spectra up to 4 s (Fig. S5†). The third component (EAS3) is long-lived (*k*_3_ = (3.0 ± 0.6) × 10^−3^ s^−1^) and has a slightly higher *ν*_CO_ (2006, 1900 cm^−1^) compared to EAS2 which is attributed to RuC2Re(COOH), which was consistent with the TR-IR spectrum at 5 min after excitation. This species is attributable to a carboxylate–ester complex RuC2Re(CO-TEOA) with the [Re^I^(diimine)(CO)_3_{C(O)OC_2_H_4_N(C_2_H_4_OH)_2_}] unit (eqn (5)). The identification of this additional intermediate is described in detail below. These results indicated that a part of the photochemically produced RuC2Re(COOH) did not return directly to RuC2Re but was converted to another long-lived complex RuC2Re(CO-TEOA). Because the bleach of the band intensity of EAS3 was approximately 60% of that of EAS2, approximately 60% of the produced RuC2Re(COOH) was converted to RuC2Re(CO-TEOA) in the TR-IR measurement. 
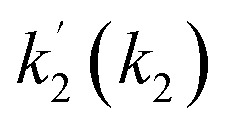
 is a rate constant that considers both the reaction of RuC2Re(COOH) directly back to RuC2Re and the conversion to the third component (RuC2Re(CO-TEOA)).5
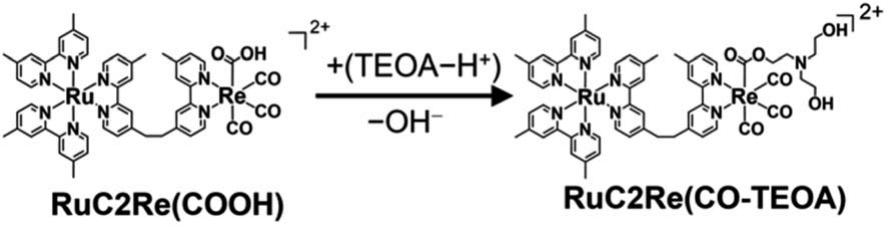


Notably, during the TR-IR measurements described in this section, RuC2Re was stable. Fig. S7† shows the FT-IR spectra of the reaction solution measured after every 10 laser irradiations (*e.g.*, the red and purple lines are the FT-IR spectra after the irradiation of pulses 10 and 60 times, respectively). The HCO_3_^−^ (1662 cm^−1^; Fig. S7a†) and free CO (2130 cm^−1^; Fig. S7b†) clearly increased by the laser irradiation; however, only a very small amount (<5% based on RuC2Re used) of RuC2Re(CO-TEOA) (2006, 1900 cm^−1^) was accumulated even after 60 laser pulses. Thus, neither the decomposition of complexes nor the accumulation of intermediates affected the measurement, *i.e.*, almost all of photoexcited complexes returned to the starting complex RuC2Re within the pulse interval in the TR-IR measurements (10 min).

### FT-IR measurements during and after steady-state light irradiation

**Fig. 4:**
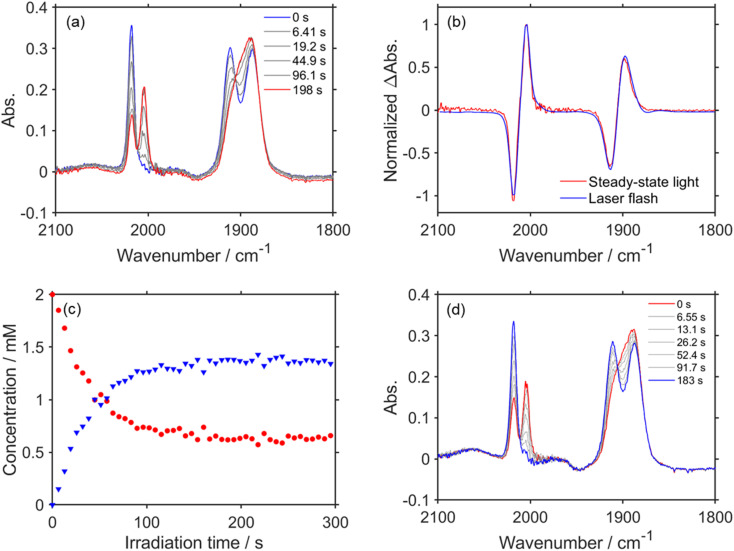
(a) FT-IR spectra of CO_2_ saturated DMSO–TEOA (5 : 1 v/v) solution containing RuC2Re (2.0 mM) and BIH (0.1 M) during steady-state light at 480 nm; *d* = 0.5 mm. (b) Normalized TR-IR spectrum at 3.0 s after laser flash (blue) and FT-IR spectrum after steady-state light irradiation for 198 s (red). (c) Concentration changes of RuC2Re (red) and RuC2Re(COOH) (blue) during steady-state light irradiation. (d) FT-IR spectra of CO_2_ saturated DMSO–TEOA (5 : 1 v/v) solution containing RuC2Re (2.0 mM) and BIH (0.1 M) after steady-state light irradiation (*λ* = 480 nm) for 2 min.

We measured the FT-IR spectra of a CO_2_ saturated DMSO–TEOA (5 : 1 v/v) solution containing RuC2Re (2.0 mM) and BIH (0.1 M) during steady-state light irradiation at 480 nm (Fig. 4a). The peaks at 2018, 1911, and 1888 cm^−1^ derived from RuC2Re decayed and the new peaks at 2004 and 1898 cm^−1^ appeared with isosbestic points at 2012 and 1906 cm^−1^. Because the difference spectrum between the FT-IR spectra before and during irradiation (Fig. 4b) was quite similar to the TR-IR spectrum at 3.0 s after the laser flash (Fig. 1b), the peaks that appeared were mainly attributed to RuC2Re(COOH). The concentration change of RuC2Re and RuC2Re(COOH) during the steady-state light irradiation (Fig. 4) was evaluated from the *ν*_CO_ peak derived from symmetry stretching vibration areas corresponding to each of the complexes; the peaks could be separated by curve fitting using a linear combination of the Gaussian function and Lorentzian functions (Fig. S8†). After ∼100 s of irradiation, the concentrations of RuC2Re and RuC2Re(COOH) reached a photo-stationary state with the ratio of RuC2Re to RuC2Re(COOH) ≈ 1 : 2, *i.e.*, both production and consumption rates of RuC2Re(COOH) became equal during the irradiation (Fig. 4c). After the irradiation, the peaks of RuC2Re(COOH) decayed and those of RuC2Re recovered (Fig. 4d). This spectral change could be fitted with a single exponential function, and the rate constant was 0.025 s^−1^ after the solution was irradiated for 120 s and then irradiation was stopped (Fig. S9†). This value is close to 
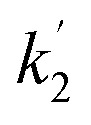
 = (4 ± 1) × 10^−2^ s^−1^ (and *k*_2_ = (3.1 ± 0.8) ×10^−2^ s^−1^) obtained by the TR-IR measurements, which is the rate constant of the subsequent reaction of RuC2Re(COOH) (Fig. 3 and S5†). These results clearly indicate that RuC2Re(COOH) is formed as the intermediate in the photocatalytic reaction of CO_2_ reduction using a steady-state light source. Although the long-lived component RuC2Re(CO-TEOA) observed in the TR-IR measurements was not clearly observed in the steady-state irradiation owing to the low SN ratio, the shoulder peak at approximately 2005 cm^−1^ remained even at 183 s after the light irradiation stopped (Fig. 4d). Furthermore, the decay rate slowed with the increasing period of light irradiation (*k* = 0.027 s^−1^ (1 min), 0.025 s^−1^ (2 min), 0.023 s^−1^ (4 min)) (Fig. S9†). This indicates that the long-lived component accumulates with an increase in the light irradiation period. These results suggest that RuC2Re(CO-TEOA), which is longer-lived than RuC2Re(COOH), gradually accumulated during the steady-state light irradiation.

### UHPLC analysis of photocatalytic reaction solutions

**Fig. 5:**
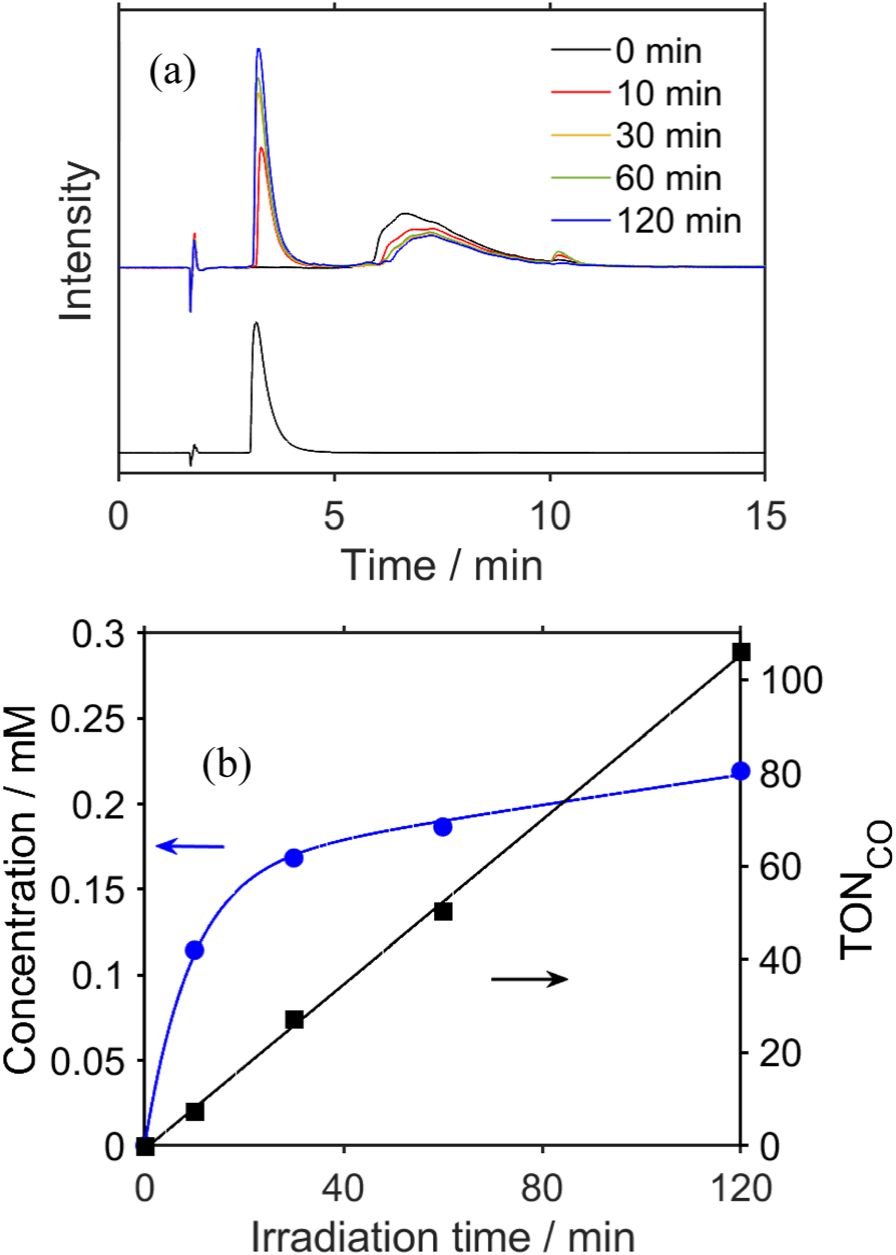
(a) UHPLC chromatograms of (top) a CO_2_ saturated DMSO–TEOA (5 : 1 v/v) solution containing RuC2Re (0.5 mM) and BIH (0.1 M) before and after irradiation at 490–620 nm (*λ*_max_ = 530 nm) for various irradiation times; the chromatogram of a DMSO–TEOA (5 : 1 v/v) solution containing RuC2Re(CO) is shown at the bottom (ODS; eluent: MeOH–KH_2_PO_4_ buffer (pH = 5.9); detection wavelength *λ*_det_ = 460 nm). (b) Concentration change of the accumulated RuC2Re(CO-TEOA), which was observed as RuC2Re(CO) in the UHPLC analysis (blue) and TON of CO formation (black) during irradiation.

To clarify the structure of the long-lived intermediate during the steady-state irradiation, the photocatalytic reaction solutions were analyzed using ultra-high-performance liquid chromatography (UHPLC). Fig. 5a shows the chromatograms of the photocatalytic reaction solutions containing RuC2Re (0.5 mM) and BIH (0.1 M) before and after irradiation at *λ*_ex_ = 490–620 nm under a CO_2_ atmosphere. The broad peak observed at 6–10 min of retention time is attributed to RuC2Re. After the light irradiation, this peak decreased and a new peak appeared at 3.3 min, which is attributed to a tetracarbonyl complex RuC2Re(CO) that has a *fac*-[Re^I^(diimine)(CO)_4_]^+^ unit because of the consistency of the retention time and the absorption spectra with the synthesized RuC2Re(CO) (Fig. S10†). After irradiation for 2 h, the accumulation of RuC2Re(CO) in the reaction solution was saturated, and, in this stage, approximately 45% of RuC2Re was converted to RuC2Re(CO) (Fig. 5b). Notably, RuC2Re(CO) is fully converted to the carboxylate–ester complex RuC2Re(CO-TEOA) in the DMSO–TEOA (5 : 1 v/v) solution (eqn (6)) as presented below. Therefore, in the weak acidic eluent (pH = 5.9), RuC2Re(CO) is recovered from RuC2Re(CO-TEOA) during the UHPLC analysis, which is the backward reaction of eqn (6). Although Re(i) tetracarbonyl complexes have been proposed as intermediates in the photocatalytic reduction of CO_2_ in various systems using *fac*-Re(diimine)(CO)_3_L type complexes,^26–28,32,62^ to the best of our knowledge, this is the first experimental identification of this intermediate in the photocatalytic reaction for CO_2_ reduction.6
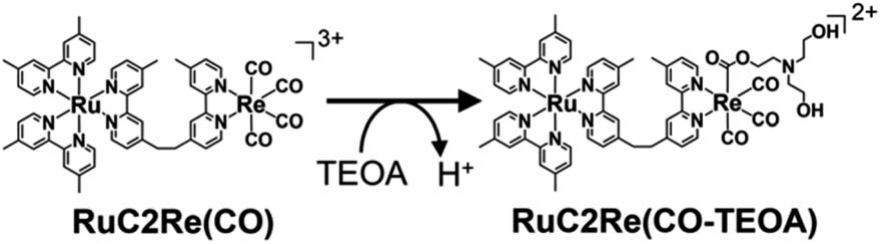


**Fig. 6:**
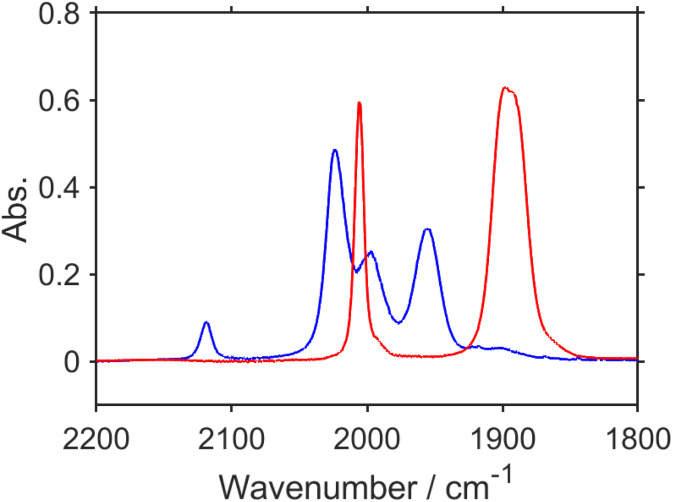
FT-IR spectra of RuC2Re(CO) (3 mM) measured in a DMSO (blue) or DMSO–TEOA (5 : 1 v/v) (red) solution; *d* = 0.5 mm.

Kubiak *et al.* reported that the one-electron reduction of the Re(i) diimine tetracarbonyl complex caused the rapid release of one of the CO ligands from the reduced complex.^62^ However, a significant amount of “RuC2Re(CO)” was detected in the UHPLC analysis of the photocatalytic reaction solution as described above even though the Re(i) diimine tetracarbonyl complexes could be easily reduced compared to the Re(i) diimine tricarbonyl complexes owing to the electron withdrawing properties of the CO ligand, *e.g.*, *E*([Re(dmb)(CO)_4_]^+^/[Re(dmb˙^−^)(CO)_4_]) = – 1.44 V *vs.* Ag/AgNO_3_ which is +160 mV more positive than the first reduction potential of Re (Fig. S11†).^46^ This accumulation is understandable because, in the photocatalytic reaction solution, the produced RuC2Re(CO) rapidly reacts with TEOA to yield RuC2Re(CO-TEOA) (eqn (6)) with a reduction potential that is more negative than that of RuC2Re(CO) as described below. RuC2Re(CO) showed four vibration bands of the CO ligands at 2119, 2024, 1997, and 1955 cm^−1^ in DMSO (Fig. 6, blue line). In the DMSO–TEOA (5 : 1 v/v) solution, on the other hand, these vibration bands were not observed at all, and another spectrum attributable to a tricarbonyl Re(i) complex was observed at *ν*_CO_ = 2006, 1895 (br) cm^−1^ (Fig. 6, red line), with a spectral change that is attributed to the addition of the deprotonated TEOA to one of the CO ligands of RuC2Re(CO) producing RuC2Re(CO-TEOA). Several similar reactions were reported, in which the nucleophilic attack of some bases such as OH^−^ and OMe^−^ to tetracarbonyl Re(i) complexes efficiently proceeded to form the corresponding carboxylate (ester) complexes.^39,63^ The addition of TEOA to the CO ligand of the [Ru(diimine)_2_(CO)_2_]^2+^-type complexes was also reported.^64,65^ It should be noted that RuC2Re(CO-TEOA) has the same *ν*_CO_s as those of the long-lived intermediate observed by the TR-IR measurement (Fig. 1b and 6). This also supports the observation that the long-lived intermediate in the TR-IR measurement is RuC2Re(CO-TEOA). It is expected that most of the produced RuC2Re(COOH) is converted to RuC2Re or RuC2Re(CO-TEOA) during the time (1–2 min) between the stopping of the light irradiation and the start of the UHPLC measurement because the lifetime of RuC2Re(COOH) is *k*_2_^−1^ ∼ 30 s in the dark.

The bimolecular rate constant of the addition of TEOA to RuC2Re(CO) was determined to be 4.56 ± 0.03 M^−1^ s^−1^ using the stopped-flow method used for mixing the DMSO–TEOA (5 : 2 v/v) solution ([TEOA] = 2.52 M) with the same volume of DMSO solution containing RuC2Re(CO) (0.1 mM), where the addition of TEOA to RuC2Re(CO) caused a red shift of the MLCT absorption band of the Re catalyst unit (Fig. S12†). Thus, the reaction rate of this addition reaction is much faster than that of the subsequent reactions of RuC2Re(COOH) and RuC2Re(CO-TEOA).

The cyclic voltammogram of the model mononuclear complex, *fac*-[Re(dmb)(CO)_3_{COOCH_2_CH_2_N(CH_2_CH_2_OH)_2_}] (Re(CO-TEOA)) first showed a reduction potential at *E*_1/2_ = −1.72 V *vs.* Ag/AgNO_3_ (Fig. S13†), which was −280 mV more negative compared to that of [Re(dmb)(CO)_4_]^+^ (*E*_1/2_ = −1.44 V, Fig. S11†). The reversibility of the first reduction of Re(CO-TEOA) decreased at a slower scan rate, suggesting that the reduction of Re(CO-TEOA) triggers the change of the carboxylate ester ligand, which releases CO (eqn (7)).7
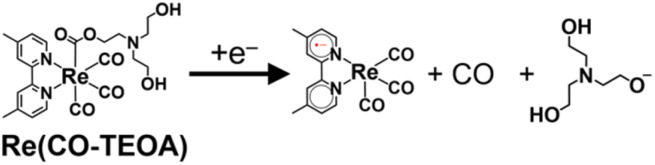


Fig. S14† shows the differential pulse voltammogram (DPV) of RuC2Re(CO-TEOA). Five reduction waves were observed at *E*_p_ = −1.48, −1.60, −1.69, −1.80 and −2.03 V *vs.* Ag/AgNO_3_. The most positive and smallest wave is attributed to the reduction of the solvent complex that is produced by the slow dissociation of the carboxylate ester ligand from RuC2Re(CO-TEOA) when preparing for the DPV measurement. The second, fourth, and fifth peaks are attributed to the Ru unit, and only the third peak is derived from the Re carboxylate ester unit, which is close to the first reduction potential of Re(CO-TEOA). Therefore, electron transfer from the one-electron reduced Ru unit to the Re carboxylate ester unit is an endergonic process. The added electron was mainly localized in the Ru unit but not in the Re unit in the one-electron reduced RuC2Re(CO-TEOA). This is the main reason why RuC2Re(CO-TEOA) is relatively stable during the photocatalytic reactions. To confirm the CO production from RuC2Re(CO-TEOA), a DMSO–TEOA (5 : 1 v/v) solution containing RuC2Re(CO-TEOA) (0.5 mM) and BIH (0.1 M) was irradiated with 490–620 nm LED light (*λ*_max_ = 530 nm) under Ar; after 5 min irradiation, RuC2Re(CO-TEOA) decreased by 23% and the same amount of CO was produced. The decay of RuC2Re(CO-TEOA) was clearly accelerated compared to that in the dark, and its rate increased under irradiation with higher light intensity (Fig. S15†). Although, therefore, the added electron is mainly localized in the Ru unit, the reductive acceleration of CO loss from the one-electron reduced RuC2Re(CO-TEOA) more rapidly proceeds compared to that from RuC2Re(CO-TEOA) itself.

## Discussion

In the TR-IR experiments, the photons were irradiated only for 7 ns and then the following processes occurred without visible-light irradiation. Because the light source used for the actual photocatalytic reaction experiments is not the pulse laser but a steady-state light source such as an LED lamp, light is continuously irradiated to the solution during the photocatalytic reaction. Therefore, the following processes after the excitation of the photosensitiser unit could be partially different between the TR-IR measurements and actual photocatalytic reactions. From this viewpoint, we first focus on the reaction mechanism of the photocatalytic CO_2_ reduction in the TR-IR measurements, and then evaluate its difference from that in the actual photocatalytic reactions.

### Reaction mechanism of CO_2_ reduction under the TR-IR measurement conditions

The rapid scan FT-IR measurements with laser flash photolysis clarified the following.

**Scheme 3:**
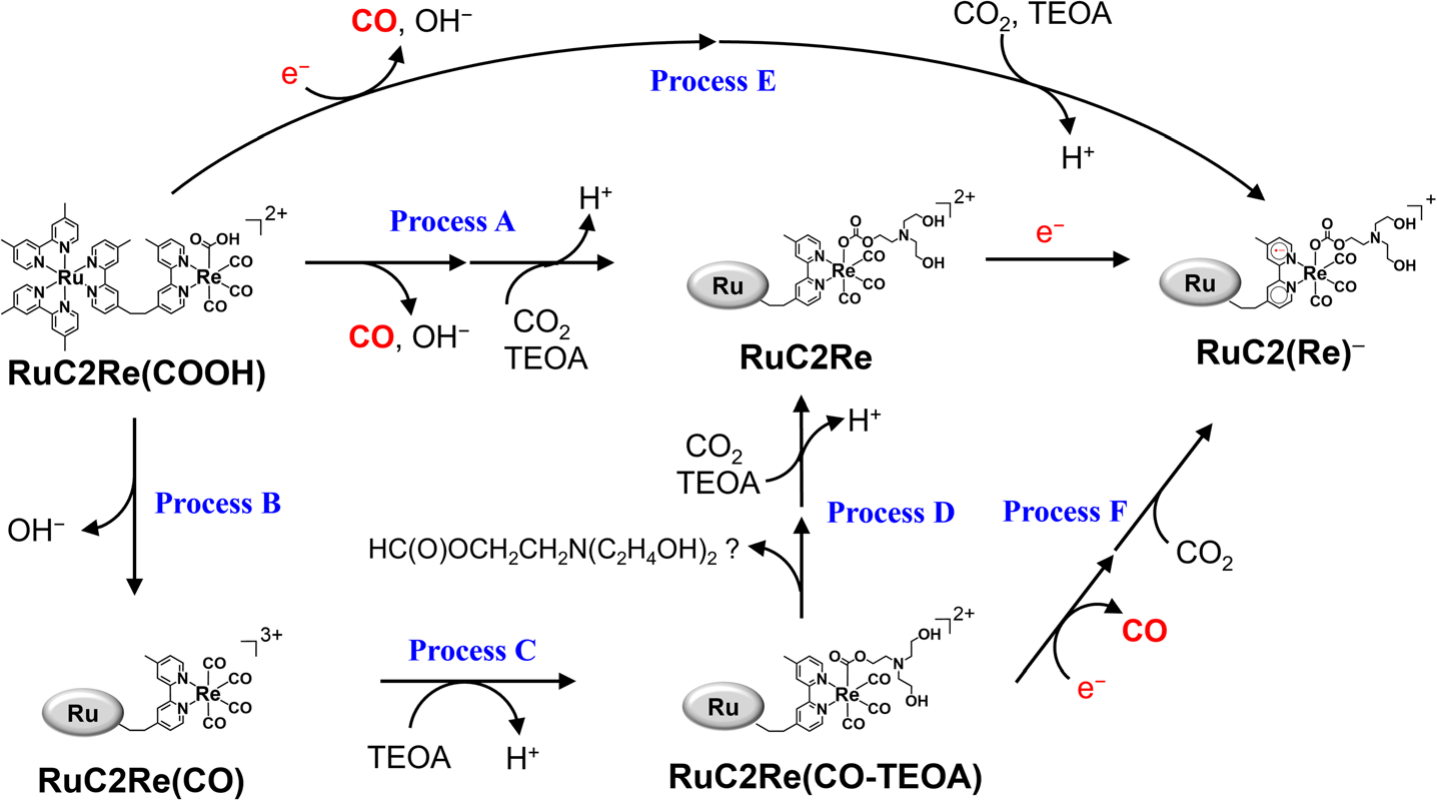
Subsequent processes of RuC2Re(COOH).

(i) OERS RuC2(Re)^−^ was quantitatively converted to the carboxylic acid intermediate RuC2Re(COOH). The isotope experiments clearly indicated that the carbon source of the C=O group of the carboxylic acid ligand in RuC2Re(COOH) is CO_2_. To the best of our knowledge, this is the first experimental detection of a Re(i) carboxylic acid complex as an intermediate that is produced by two-electron reduction of the *fac*-[Re(diimine)(CO)_3_L]^*n*+^-type complex during photocatalytic reactions.^25,27,28,35,36,38–40^ The rate of this conversion reaction linearly depends on the concentration of RuC2(Re)^−^ with a rate constant of *k*_1_ = 1.8 s^−1^.

(ii) RuC2Re(COOH) changed the structure with *k*_2_ = (3.1 ± 0.8) × 10^−2^ s^−1^. Approximately 40% of RuC2Re(COOH) returned to the starting complex RuC2Re with the release of CO and OH^−^ and the addition of CO_2_ and a deprotonated TEOA in the dark reaction (Process A in Scheme 3). The pseudo-first order rate constant of the CO_2_ capture reaction by the deprotonated TEOA coordinated complex was determined to be 5.2 s^−1^ (Fig. S21†) under a 100% CO_2_ atmosphere. Because this rate of the CO_2_ capture reaction is two orders of magnitude faster than *k*_2_, the rate determining step of Process A is not by the coordination of the deprotonated TEOA and CO_2_ insertion to Re–O bond but by the release of CO and OH^−^ from RuC2Re(COOH).

(iii) Approximately 60% of RuC2Re(COOH) was converted to another long-lived intermediate RuC2Re(CO-TEOA) that was produced by the addition of TEOA to the tetracarbonyl complex RuC2Re(CO) (Processes B and C in Scheme 3). It was deduced that another CO_2_ molecule attacks the O atom of the OH group of *fac*-[Re^I^(dmb)(CO)_3_(COOH)] to form [Re(dmb)(CO)_4_]^+^ and HCO_3_^−^.^41^ During the TR-IR measurements, RuC2Re(CO-TEOA) slowly but quantitatively returned to RuC2Re with *k*_3_ = (3.0 ± 0.6) × 10^−3^ s^−1^ (Process D).

### Conversion reaction mechanism from RuC2(Re)^−^ to RuC2Re(COOH)

RuC2Re required two electrons to be converted to RuC2Re(COOH). In other words, the one-electron reduced species RuC2(Re)^−^ or the subsequent intermediate made from RuC2(Re)^−^ received one more electron to produce RuC2Re(COOH), which is the second reduction process in the photocatalytic cycle for CO_2_ reduction. Because the rate of this reductive conversion reaction linearly depended on the concentration of RuC2(Re)^−^ with *k*_1_ = 1.8 ± 0.1 s^−1^ (Fig. 1 and S5†), the disproportionation reaction of two molecules of RuC2(Re)^−^ was excluded from the subsequent processes of RuC2(Re)^−^. Under the experimental conditions of the TR-IR measurements using the ∼7 ns laser pulse, the excitation of RuC2(Re)^−^ and the intermediate could not proceed at a time period of sub-seconds after the laser flash because the interval of the laser pulses was 10 min. Although BI˙ has a strong reducing power, it is not the main electron donor to RuC2(Re)^−^ and/or the intermediate because, in the TR-IR measurements, all the produced BI˙ passed an electron to RuC2Re on the microsecond time scale.^46,66^ Therefore, the main second-electron donor to the intermediate (but not to another molecule of RuC2(Re)^−^, *i.e.*, not disproportionation of RuC2(Re)^−^, as described above) was only RuC2(Re)^−^ (and partially (Ru)^−^C2Re) in the TR-IR experiments. This is strongly supported by the fact that only a half of the RuC2Re(COOH) formed compared to the amount of the decreased RuC2(Re)^−^ and, simultaneously, a half of the bleaching band attributed to the recovery of RuC2Re was observed. Based on these results and investigations, we can conclude that the rate determining step in the production processes of RuC2Re(COOH) is not the electron transfer (mainly from RuC2(Re)^−^) to another intermediate RuC2Re(X) (eqn (9)), but the structure change process of RuC2(Re)^−^ to RuC2Re(X) (eqn (8)). After this structure change process, RuC2Re(X) accepts one electron from RuC2(Re)^−^ or (Ru)^−^C2Re for conversion to RuC2Re(COOH), which is a faster process compared to the structure change.8
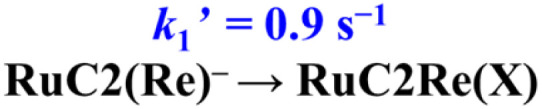
9
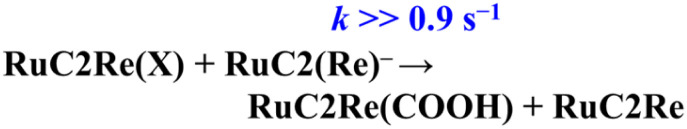


Recently, we reported that the intermediate Re(X) produced from the one-electron-reduced mononuclear Re complex Re^−^ has a more positive reduction potential than that of Re (−1.6 V *vs.* Ag/AgNO_3_) and cannot be reduced by the reduced photosensitiser with a reduction potential that is more positive than *E*^red^_1/2_ = −1.4 V *vs.* Ag/AgNO_3_.^67^ Therefore, the second electron-transfer reaction from RuC2(Re)^−^ to RuC2Re(X) is exergonic and, therefore, rapid. Because the decrease of RuC2(Re)^−^ occurs *via* two processes (eqn (8) and (9)), the actual rate constant of the structure change from RuC2(Re)^−^ to RuC2Re(X) is a half of *k*_1_ (the decay constant of RuC2(Re)^−^), *i.e.*, 
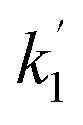
 = 0.9 s^−1^ (eqn (8)). To obtain information on this intermediate RuC2Re(X), we conducted similar TR-IR experiments in a CO_2_ saturated DMSO–TEOA (5 : 1 v/v) solution containing NH_4_PF_6_ (20 mM) as the proton source (Fig. S16†). Under these conditions, the converted reaction of RuC2(Re)^−^ to RuC2Re(COOH) (*k*_1_ = 2.7 s^−1^) was approximately 1.5 times faster than that in the absence of NH_4_PF_6_. This result suggested that protons are involved in the subsequent process of RuC2(Re)^−^ converting to RuC2Re(X).

**Scheme 4:**
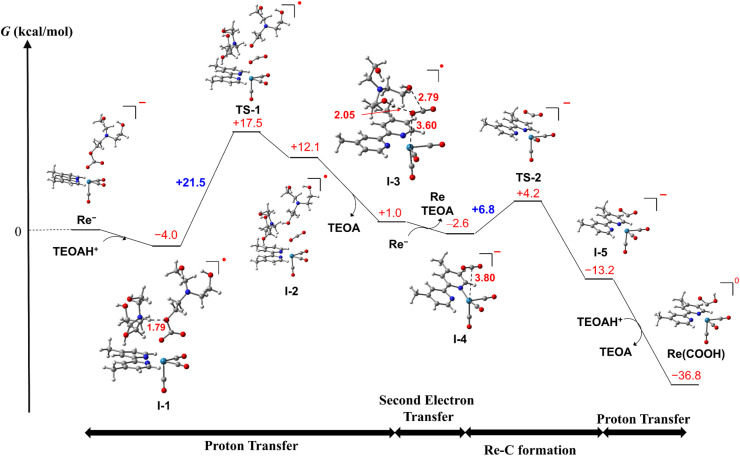
Computed free energy profile for the conversion reaction from the OERS of *fac*-Re(dmb)(CO)_3_{OC(O)OCH_2_CH_2_N(CH_2_CH_2_OH)_2_} (Re^−^) to *fac*-Re(dmb)(CO)_3_(COOH) (Re(COOH)). Re–C bond formation occurs after the second electron reduction.

We should not be able to obtain the spectral data of RuC2Re(X) directly in the photocatalytic reaction because the concentration of RuC2Re(X) should be very low owing to the subsequent rapid reduction reaction (eqn (9)). Therefore, we evaluated the mechanism of the conversion reaction of the model mononuclear complex Re^−^ using the DFT calculations based on the experimental results as described above (Schemes 4 and S1†). In the initial process, a hydrogen bond forms between the oxygen atom of the carbonate ester ligand of Re^−^ and a protonated TEOA (tertiary ammonium ion: H–O = 1.79 Å, Δ*G*° = −4.0 kcal mol^−1^), *i.e.*, formation of I-1, and then the proton transfer from the N^TEOAH^+^^ to the O^TEOA^ atom of the carbonate ester ligand that coordinates to the Re center to cleave the C–O^TEOA^ bond of the carbonate ester ligand *via*TS-1 (Δ*G*^‡^ = +21.5 kcal mol^−1^). As a result, an intermediate I-2 is formed (Δ*G*° = +16.1 kcal mol^−1^) and is favorably converted to I-3 (Δ*G*° = −11.1 kcal mol^−1^), in which CO_2_, previously involved in the carbonate ester bond, forms a weak coordination bond with the Re center *via* one of the O atoms (Re–O = 3.60 Å, ∠O=C=O = 177°) and the dissociated TEOA interacts with CO_2_ (O–C = 2.79 Å, H–O = 2.05 Å). There are two plausible subsequent processes of I-3 as follows.

(1) As shown in Scheme 4, I-3 favorably accepts an electron before the Re–C bond formation to form the two-electron reduced species I-4 (Δ*G*° = −3.6 kcal mol^−1^ in the calculation using Re^−^ as the electron donor), in which the Re center is weakly coordinated with CO_2_*via* the C atom (Re–O = 3.80 Å, ∠O=C=O = 178°). I-4 is readily converted to the C-coordinated CO_2_ adduct I-5*via*TS-2 (Δ*G*° = −10.6 kcal mol^−1^, Δ*G*^‡^ = +6.8 kcal mol^−1^). This reaction is thermodynamically more favorable than the CO_2_ cleavage in I-4, *i.e.*, the dissociation of CO_2_ from the Re center (Δ*G*° = −3.3 kcal mol^−1^).

(2) The formation processes of I-3 are the same as in Scheme 4. As shown in Scheme S1,†I-3 is converted to Re(dmb)(CO)_3_(CO_2_) (I-4′) with the Re–C bond *via*TS-2′ (Δ*G*° = +3.4 kcal mol^−1^, Δ*G*^‡^ = +7.1 kcal mol^−1^), and then I-4′ accepts an electron, forming the two-electron reduced species I-5 (Δ*G*° = −17.7 kcal mol^−1^ (=−770 meV)).

On one hand, the calculated reduction potential of I-4′ is 770 mV more positive than that of Re. On the other hand, because the calculated reduction potential of I-3 is +160 mV more positive than that of Re, which is consistent with the experimental results described above,^67^ the mechanism shown in Scheme 4 is more reliable.

Protonation of I-5 produces the carboxylic acid complex Re(COOH) (Δ*G*° = −23.6 kcal mol^−1^). These calculations suggest that the rate-determining step in the conversion reaction from Re^−^ to Re(COOH) is the proton transfer *via*TS-1, which is consistent with the experimental observations, *i.e.*, the addition of the proton source accelerated the conversion rate of RuC2(Re)^−^ to Re(COOH). Based on these results and investigations, it can be inferred that the structure of the Re unit in the experimentally unobservable intermediate RuC2Re(X) that accepts the second electron is the CO_2_ coordination complex I-3 (Scheme 4).

### Electron donor for the second reduction process and role of RuC2Re(CO-TEOA) in the actual photocatalytic CO_2_ reduction reaction

Fig. 5b shows that the concentrations of the metal complexes such as RuC2Re and RuC2Re(CO-TEOA) drastically changed during the photocatalytic reactions for the CO_2_ reduction using the steady-state light. Their concentrations were different from that observed in the TR-IR measurement, and changed depending on the light intensity of the irradiation. The starting complex RuC2Re and the relatively stable intermediates, *i.e.*, RuC2Re(COOH) and RuC2Re(CO-TEOA), absorb light during the photocatalytic reactions whereas, in the TR-IR measurements using the pulse laser, only RuC2Re is excited even though the light flux of the laser pulse is much higher than that of the steady-state irradiation. Therefore, we reconsidered the role of the accumulated intermediates as the “external” redox photosensitisers which might supply an electron to the intermediate RuC2Re(X) in the second reduction process, and the CO release mechanism as well.

The detected RuC2Re(CO-TEOA) could be one of the intermediates that produce CO during the photocatalytic reaction. The TR-IR measurement indicated that RuC2Re(CO-TEOA) slowly returned to RuC2Re under dark conditions with *k*_3_ = (3.0 ± 0.6) × 10^−3^ s^−1^ (Fig. 1b and 3). To confirm CO production by this reaction (Process D in Scheme 3), the gas and liquid phases of a sealed 11 mL sample tube, which included a CO_2_ saturated DMSO–TEOA (5 : 1 v/v) solution (1 mL) containing RuC2Re(CO-TEOA) (0.5 mM) and BIH (0.1 M), were analyzed by GC and UHPLC respectively. In the dark, no CO was formed even though RuC2Re(CO-TEOA) was converted to RuC2Re. Although we could not identify the product from the carboxylate ester ligand, it could be the carboxylate ester (formate ester) itself, N(CH_2_CH_2_OH)_2_(CH_2_CH_2_OCHO).

How about the photochemical reaction of RuC2Re(CO-TEOA) during the photocatalytic reaction? The decay of RuC2Re(CO-TEOA) was accelerated during irradiation in the presence of BIH compared to that in the dark; the photochemical reaction rate increased under irradiation with a higher light intensity (Fig. S15†). The amount of CO detected was similar to the decreased amount of RuC2Re(CO-TEOA) in the photochemical reaction. These results indicate that the OERS of RuC2Re(CO-TEOA) ([RuC2Re(CO-TEOA)]^−^), which was produced *via* photochemical electron transfer from BIH to the excited Ru photosensitiser unit of RuC2Re(CO-TEOA) or reduction of ground state RuC2Re(CO-TEOA) by BI˙, quantitatively releases CO (Process F in Scheme 3). It should be noted that this photochemical CO production from RuC2Re(CO-TEOA) (∼0.052 min^−1^) was much slower than the CO formation during the photocatalytic CO_2_ reduction using RuC2Re with the same light intensity (TOF_CO_ = 0.88 min^−1^, Fig. 5). As shown in Fig. 5b, a certain amount of RuC2Re (the initial concentration was 0.5 mM) was converted to RuC2Re(CO-TEOA) in the initial stage of the photocatalytic reduction; 0.22 mM RuC2Re(CO-TEOA) was accumulated in the photocatalytic reaction solution after irradiation for 120 min. The concentration of RuC2Re(CO-TEOA) rapidly increased in the first stage of the photocatalytic reaction, and then almost stabilized until 120 min of irradiation. A comprehensive evaluation of these results indicates that the CO release from [RuC2Re(CO-TEOA)]^−^ during the photocatalytic reaction (Processes B, C and F in Scheme 3) is not the primary pathway of the photocatalytic CO_2_ reduction using RuC2Re because its reaction rate was too slow as described above (Fig. S15†) and the continuous and stable formation of CO was already observed 10 min after the irradiation started, and then continued until 2 h of irradiation even though the concentrations of RuC2Re and RuC2Re(CO-TEOA) were changed in the initial stage of the photocatalytic reaction (Fig. 5). Therefore, the main process of the subsequent reactions of RuC2Re(COOH) in the photocatalytic reactions was the direct formation of CO from RuC2Re(COOH) (Process A).

It is reasonable to deduce that not only RuC2(Re)^−^ but also [RuC2Re(CO-TEOA)]^−^ worked as the electron donor because a considerable amount of RuC2Re(CO-TEOA) was accumulated in the reaction solution during the photocatalytic reactions (Fig. 5). The excited state of RuC2Re(CO-TEOA) had a lifetime that was long enough and the excited state of its Ru unit was efficiently quenched by BIH. Because the reduction potential of the Re unit of RuC2Re(CO-TEOA) is 90 mV more negative than that of the Ru unit, the added electron to RuC2Re(CO-TEOA) is mainly localized at the Ru unit. The role of [RuC2Re(CO-TEOA)]^−^ as the electron donor not only to RuC2Re(X) but also to the starting complex RuC2Re causes a longer lifetime of RuC2Re(CO-TEOA) in the steady-state irradiation. Thus, the accumulated RuC2Re(CO-TEOA) not only works as the precursor of CO formation after its reduction but also as an “external” redox photosensitiser in the photocatalytic reaction. This was the main reason why the larger amount of RuC2Re(CO-TEOA) was accumulated during the steady-state irradiation (Fig. 5).^46^

On the other hand, BI˙ should not be the main electron donor of the second reduction in the photocatalytic reactions because the concentration of the intermediate RuC2Re(X) was much lower than that of the other complexes such as RuC2Re, RuC2Re(COOH) and RuC2Re(CO-TEOA) that could accept electrons from BI˙ during the photocatalytic reactions. The excitation of RuC2(Re)^−^ and/or RuC2Re(X) followed by reductive quenching with BIH had very low possibilities as a second reduction process because of the low flux of photons and low concentration of RuC2(Re)^−^ and RuC2Re(X) under the photocatalytic reaction conditions. For example, in the reported quantum yield measurements of RuC2Re,^46^ the number of photons absorbed by the Ru photosensitiser unit was 3.7 × 10^−9^ einstein s^−1^ (480 nm), and the amount of the Ru photosensitiser unit was 0.2 μmol in the solution. This indicates that one Ru photosensitiser unit can absorb one photon every 53 s on average. Therefore, the lifetimes of RuC2(Re)^−^ (*k*_1_^−1^ = 0.56 s) and RuC2Re(X) (≪0.56 s) are too short for these intermediates to absorb a photon. Additionally, excitation of the Ru unit of RuC2(Re)^−^ caused not only reductive quenching by BIH but also intramolecular reductive quenching from the one-electron reduced Re unit to the excited state of the Ru unit, which was followed by rapid intramolecular back electron transfer.^68^

### CO release process from RuC2Re(COOH)

RuC2Re(COOH) exhibited a comparatively prolonged lifetime in the dark and had two conversion processes (Processes A and B in Scheme 3). The dominant process for CO production is Process A (*k* = 0.03 s^−1^ in the dark) as described above in the TR-IR measurements. If the CO-release process from RuC2Re(COOH) was only the dark reaction, it would become the rate-determining step of the photocatalytic CO_2_ reduction under higher light intensity. However, under irradiation using a strong LED-light (*λ*_ex_ = 440–600 nm), which was the strongest light intensity in our laboratory, the turnover frequency of the photocatalytic CO formation (TOF_CO_) was up to *k* = 0.4 s^−1^ (Fig. S17†), which was much faster than the CO-release reactions of RuC2Re(COOH) in the dark. It is noteworthy that the rate of the subsequent reaction of RuC2Re(COOH) (Process A) (*k* = 0.03 s^−1^) was measured in the TR-IR experiments after laser irradiation or just after cutting steady-light irradiation (Fig. 1b and S9†), particularly, without irradiation. The “unexpected” faster reaction rates of CO formation in the photocatalytic reaction originated from photochemical reduction and/or reduction of the accumulated RuC2Re(COOH) (Process E). The accumulation of RuC2Re(COOH) enabled not only the photochemical reduction of RuC2Re(COOH) but also its reduction by BI˙ and/or the one-electron-reduced form of RuC2Re(CO-TEOA). It was deduced that the reduced Re^0^ carboxylic acid complexes [Re^0^(bpy)(CO)_3_(COOH)]^−^ were converted to the corresponding [Re^0^(bpy)(CO)_4_]^0^ and released CO.^25,32,62^

Fig. S18† shows the CV of Re(COOH) in an Ar purged DMSO–TEOA (5 : 1 v/v) solution. Re(COOH) exhibits a chemically irreversible reduction wave at *E*_1/2_ = −1.73 V *vs.* Ag/AgNO_3_ and a reoxidation wave at *E*_p_ = −1.53 V, which is assigned to the one-electron reduced DMSO and/or TEOA coordinated complex produced after the CO release reaction from the one-electron reduced Re(COOH). From these results and investigations, under high light-intensity conditions, the reduction of RuC2Re(COOH) and the subsequent CO release from the reduced complex was one of the main CO-formation pathways in the photocatalytic reaction. Fig. S19† shows the UV-vis absorption spectrum changes of a photocatalytic reaction solution containing RuC2Re (0.05 mM) and BIH (0.1 M) during steady-state light irradiation with a relatively high light intensity (*λ*_ex_ = 480 nm, 2.6 × 10^−8^ einstein s^−1^). Under these conditions, the Ru unit can absorb a photon every 11 s on average. The absorption band appeared at 515 nm in the initial stage of the photocatalytic reaction. This absorption band is assigned to the one-electron reduced species of RuC2Re, *i.e.*, the equilibrium mixture of (Ru)^−^C2Re and RuC2(Re)^−^. After more than 60 s of light irradiation, the shape of the absorption band drastically changed to that at *λ*_max_ = 510 and 535 nm, which is consistent with the absorption of the one-electron reduced species of [Ru(dmb)_3_]^2+^.^46,66^ This spectral change clearly indicates the formation of (Ru)^−^C2Re(COOH) (and partially (Ru)^−^C2Re(CO-TEOA)) in which the Re unit has a more negative reduction potentials compared to the Ru unit.^69^ Although the electron transfer from the one-electron-reduced Ru unit to the Re carboxylic acid unit is endergonic, the CO release reaction from the one-electron reduced RuC2Re(COOH) should proceed because of the irreversibility of the one-electron reduced Re carboxylic acid complex.

It should be interesting to investigate another photocatalytic system using 1-benzyl-1,4-dihydronicotinamide (BNAH) that can donate only one electron instead of BIH that donates two electrons. It was reported that the maximum TOF_CO_ was 0.078 s^−1^ when BNAH was used as the sacrificial electron donor.^70^ This was close to the rate of the CO release reaction from RuC2Re(COOH) (*k* = 0.03 s^−1^) probably because the reduction process of RuC2Re(COOH) was much slower compared to the system using BIH. These deductions clearly indicate that the rate-determining step of the photocatalytic CO_2_ reduction by the Ru(ii)–Re(i) supramolecular photocatalysts strongly depends on the reaction conditions, *i.e.*, light intensity, type of sacrificial electron donor, and concentration of the photocatalyst.

It is noteworthy that the pseudo-first-order rate constant of the CO_2_ capture reaction under the 100% CO_2_ atmosphere (5.2 s^−1^) was above 10 times faster than the highest TOF_CO_ using high light intensity (0.4 s^−1^) (Fig. S21†). This result clearly indicates that the CO_2_ capture reaction is not a rate determining step of the photocatalytic reduction of CO_2_. This is one of the reasons why the Ru(ii)–Re(i) supramolecular photocatalyst exhibits excellent photocatalytic activity even under a low-concentration CO_2_ atmosphere.^6^

## Conclusions

**Scheme 5:**
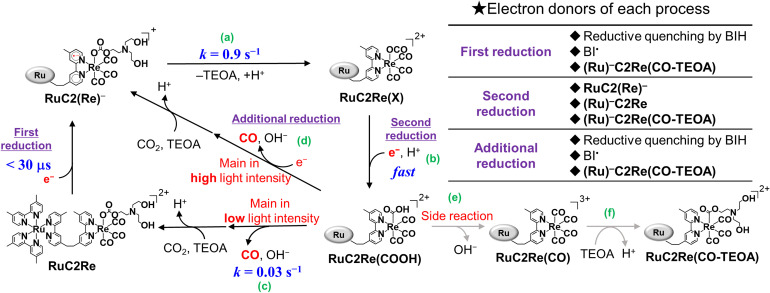
Full reaction mechanism of photocatalytic CO_2_ reduction by using RuC2Re and BIH.

The overall reaction mechanism of the photocatalytic reduction of CO_2_ using RuC2Re consisting of the [Ru(diimine)_3_]^2+^ photosensitiser and the *fac*-[Re(diimine)(CO)_3_{OC(O)OCH_2_CH_2_NR_2_}] catalyst units was elucidated as shown in Scheme 5.

The carboxylic acid complex RuC2Re(COOH) was detected as a subsequent intermediate of the one-electron reduced species (OERS) RuC2(Re)^−^ by time-resolved IR (TR-IR) measurements using rapid-scan FT-IR spectroscopy with laser flash photolysis as well as in the actual photocatalytic reaction using steady-state irradiation. The kinetic analysis of the TR-IR spectra and DFT calculations revealed that the Re unit of RuC2Re^−^ changes its structure to a CO_2_-coordinated complex (Process (a)), and then accepts an additional electron from another OERS to form RuC2Re(COOH) (Process (b)).

There were two conversion processes of RuC2Re(COOH). The main process involved the release of CO and OH^−^, and RuC2Re was recovered without forming any other long-lived intermediate (Process (c)). Under the actual photocatalytic reaction conditions, especially when the light intensity was high, RuC2Re(COOH) was reduced by photoinduced electron transfer and by BI˙ to accelerate the release of CO (Process (d)). As a side reaction, RuC2Re(COOH) released only OH^−^ to form a tetracarbonyl species, RuC2Re(CO) (Process (e)), that was rapidly converted to the carboxylate ester complex RuC2Re(CO-TEOA) by the nucleophilic attack of TEOA (Process (f)). RuC2Re(CO-TEOA) has a relatively long lifetime, but the reduction of RuC2Re(CO-TEOA) induces the release of CO from this intermediate. RuC2Re(CO-TEOA) serves not only as the precursor for CO formation but also as an external redox photosensitiser in the photocatalytic reaction.

## Data availability

All the data supporting this article have been included in the main text and the ESI.†

## Author contributions

Kei Kamogawa: most of the experiments and calculations, discussion, writing the manuscript. Yuki Kato and Takumi Noguchi: TR-IR measurements using the rapid scan FT-IR method, discussion, writing the manuscript. Koichi Nozaki: calculations, discussion. Yusuke Tamaki: some experiments, discussion. Tatsuo Nakagawa: experiments using the stopped flow method. Osamu Ishitani: planning the research, discussion, collecting the research, writing the manuscript.

## Conflicts of interest

There are no conflicts to declare.

## Supplementary Material

SC-015-D3SC06059D-s001
