# Supplementary material for: Overall reaction mechanism of photocatalytic CO2 reduction on a Re(i)-complex catalyst unit of a Ru(ii)–Re(i) supramolecular photocatalyst
Source: Chem Sci. 2023 Dec 21;15(6):2074–88. doi: 10.1039/d3sc06059d (PMC10848666; doi:10.1039/d3sc06059d)
Supplement: SC-015-D3SC06059D-s001 [file SC-015-D3SC06059D-s001.pdf]

Electronic Supplementary Material (ESI) for Chemical Science.
This journal is © The Royal Society of Chemistry 2023

Supporting information

Overall mechanism of photocatalytic CO_2 reduction on a Re(I)-complex catalyst unit of Ru(II)-Re(I) supramolecular photocatalyst

Kei Kamogawa,[1] Yuki Kato,[2]* Yusuke Tamaki,[1,†] Takumi Noguchi,[2] Koichi Nozaki,[3] Tatsuo Nakagawa,[4] Osamu Ishitani[1,5]*

[1]Department of Chemistry, School of Science, Tokyo Institute of Technology, 2-12-1-NE-2 O-okayama, Meguro-ku, Tokyo 152-8550, Japan

[2]Department of Physics, Graduate School of Science, Nagoya University, Nagoya 464-8602, Japan

[3]Department of Chemistry, Graduated School of Science and Engineering, University of Toyama, 3190, Gofuku, Toyama-shi, Toyama 930-8555, Japan.

[4]UNISOKU Co., Ltd., 2-4-3 Kasugano, Hirakata, Osaka, 573-0131, JAPAN

[5]Department of Chemistry, Graduate School of Advanced Science and Engineering, Hiroshima University, 1-3-1 Kagamiyama, Higashi-Hiroshima, Hiroshima 739 8526, Japan

Experimental Section

General procedures

The ^1H NMR spectra were measured using a JEOL ECA400II spectrometer (400 MHz). The residual protons were used as the internal standards in these measurements. The FT-IR absorption spectra were recorded using a JASCO FT-IR 6600 spectrophotometer except for the rapid scan FT-IR measurement. For the FT-IR measurement during steady-state light irradiation, a 300 W Xe lamp (Asahi Spectra MAX-303) equipped with a bandpass filter (480 nm) was used. The stopped-flow measurements were performed using a stopped-flow rapid scan spectrophotometer (RSP-2000; Unisoku Co. Ltd.). The CV and DPV measurements were performed using an ALS/CHI760E electrochemical analyzer and a solution containing Et_4NBF_4 (0.1 M) as a supporting electrolyte, a glassy carbon disc working electrode (diameter = 3 mm or 33 μm), an Ag/$AgNO_3$ (0.01 M $AgNO_3$ in DMSO) reference electrode, and a Pt wire counter electrode. The ferrocenium/ferrocene redox couple was observed at +0.18 V *vs.* Ag/ $AgNO_3$ in DMSO. The working electrode was polished using 0.05 μm alumina pastes before every measurement. *iR* compensation was performed using the positive feedback method, which was implemented using the ALS/CHI760E analyzer. The produced CO was analyzed by gas chromatography with TCD as a detector (GL Sciences GC323). The global fitting analysis for the time-dependence of the IR spectra were performed using the Glotaran program (http://glotaran.org).

Materials

Prior to its use in the measurements, DMSO was dried over 4 Å molecular sieves at room temperature before being distilled in the presence of calcium hydride at reduced pressure. Super dehydrated DMSO purchased from Fujifilm Wako Pure Chemical Corporation was used for the TR-IR measurements. TEOA was distilled under reduced pressure (<133 Pa) prior to its use. After distillation, both solvents were stored under an Ar atmosphere. Et_4NBF_4 was dried under a vacuum overnight at 100 °C prior to its use. All other reagents were of reagent-grade quality and were used without further purification. BIH and *fac*-[(dmb)$_2$Ru(bpyC$_2$bpy)Re(CO)$_3$MeCN](PF$_6$)$_2$ (**RuC2Re(MeCN)**, dmb = 4,4′-dimethyl-2,2′-bipyridine, bpyC$_2$bpy = (4′-methyl-[2,2′-bipyridine]-4-yl)-CH$_2$CH$_2$-(4′-methyl-[2,2′-bipyridine]-4-yl)) were synthesized according to the procedures reported in previous literature.[1-4] *fac*-Re(dmb)(CO)$_3$(COOH) (**Re(COOH)**) was prepared using the method for the synthesis of *fac*-Re(bpy)(CO)$_3$(COOH) described in previous studies, except that [Re(dmb)(CO)$_4$](OTf) was used as a starting material.[5, 6]

RuC2Re: **RuC2Re(MeCN)** was dissolved in DMSO, and the resulting solution was kept at room temperature in the dark under an Ar atmosphere for 4.5 h for changing the MeCN ligand of all of the added Re complexes to the DMSO ligand. After this time, TEOA was added into the solution (DMSO : TEOA = 5 : 1 v/v), which was kept under an Ar atmosphere in the dark at room temperature for a period of more than 4 h. The resulting solution was bubbled with CO_2 to give a DMSO–TEOA (5 : 1 v/v) solution containing **RuC2Re**. In the experiment under $^{13}CO_2$ atmosphere, **RuC2Re** with ^{13}C-labeled carbonate ester bonds was prepared by bubbling with $^{13}CO_2$ instead of ordinary CO_2.

RuC2Re(CO): [(dmb)2Ru(bpyC2bpy)](PF$_6$)$_2$ (bpyC2bpy = (4’-methyl-[2,2’-bipyridine]-4-yl)-CH$_2$CH$_2$-(4’-methyl-[2,2’-bipyridine]-4-yl):) (96.4 mg, 0.0856 mmol) and Re(CO)$_5$OTf (54.6 mg, 0.115 mmol) were dissolved in CH_2Cl_2 40 mL. The reaction mixture was refluxed for 3 days. After the solvent was evaporated, the residue was dissolved in H_2O-acetone (1:1 v/v) containing NaBr (0.1 M) and left for 3 h. After the acetone was evaporated, a saturated aqueous solution of NH_4PF_6 was added to the filtrate to obtain an orange precipitate. After filtration, the orange solid was purified by ion-exchange column chromatography (CM Sephadex C-25, eluent: acetone-water (1:1 v/v) containing NaBr) under dark conditions. After the acetone was removed, a saturated aqueous solution of NH_4PF_6 was added to the solution, and the precipitate was extracted with CH_2Cl_2/H_2O. The product was isolated as a red powder by recrystallization with CH_2Cl_2/Et_2O. Yield: 50.8 mg (37.8%). FT-IR (CH_2Cl_2) ν_{CO}/cm^{-1}: 2121, 2026, 2008, 1967. ^{1}H NMR (400 MHz, acetone-*d*$_6$) δ/ppm: 9.16 (d, 1H, *J*=6 Hz, γ-bpy-3), 9.11 (d, 1 H, *J*=6 Hz, δ-bpy-3), 8.81 (m, 2H, α-bpy-3, β-bpy-3), 8.68 (m, 6 H, dmb-3,3’, α-bpy-2, β-bpy-2), 7.94 (d, 1 H, *J*=6 Hz, δ-bpy-6), 7.87-7.81 (m, 6 H, dmb-6,6’, β-bpy-5, γ-bpy-6), 7.87 (d, 1 H, *J*=6 Hz, α-bpy-5), 7.56 (d, 1 H, *J* = 6 Hz, δ-bpy-5), 7.40-7.36 (m, 5 H, dmb-5,5’, γ-bpy-5), 3.37-3.29 (m, 4 H, -CH$_2$CH$_2$-), 2.68 (s, 3 H, δ-py-*CH$_3$*), 2.57 (m, 15 H, dmb-*CH$_3$*, α-py-*CH$_3$*) (Figure S20). Elemental analysis: C (39.76%), H (2.87%), and N (7.32%), calculated for $C_{52}H_{46}N_8O_4P_3F_{18}ReRu$: C (39.80%), H (2.95%), and N (7.14%).

Rapid scan time-resolved FT-IR measurement

The rapid-scan time-resolved FTIR spectra of the CO_2 saturated DMSO-TEOA (5:1 v/v) solution containing **RuC2Re** (1.0 mM) and BIH (0.1 M) in an IR cell with an optical path length of 0.1 mm were recorded on a Bruker Vertex 80 spectrophotometer (resolution: 4 cm^{-1}) with a scan velocity of 320 kHz and the acquisition mode of the single-sided forward and backward at 298 K. A Ge filter to cut infrared light at >2200 cm^{-1} (Andover, 4.50ILP-25) was placed in the infrared beam path. Two scans that consist of one lap of forward and backward scans require 42 ms with an additional interval of 6 ms. The visible pump pulse was obtained by generating the second harmonic (532 nm) of the Q-switched

Nd:YAG laser [Quanta-Ray INDI-40–10; ~7 ns full width at half-maximum (FWFM), ~50 mJ cm^{-2} $pulse^{-1}$]. One 2-scan measurement before a single flash and then forty 2-scan, fifteen 8-scan, eleven 210-scan, and twelve 840-scan measurements after the flash were carried out. The laser flash interval was 10 min.

UHPLC analysis

The DMSO-TEOA (5:1 v/v, 1 mL) solution containing **RuC2Re** (0.5 mM) and BIH (0.1 M) in 11 mL volume Pyrex test tubes were purged with CO_2 for 60 min, and then irradiated at 490–620 nm (λ_{max} = 530 nm) using an Iris-MG merry-go-round irradiation apparatus with an LED light source (CELL System Co.). Ultra-High-Performance Chromatography (UHPLC) analyses were performed using an ODS (Waters Acquity) column (150 mm × 2.1 mm i.d.), a Shimadzu DGU-20A degasser, a LC-30AD pump, a SPD-M30A UV-vis photodiode-array detector, and Rheodyne 7125 injector. The eluent was a 1:1 (v/v) mixture of methanol and a 0.05 M KH_2PO_4 buffer (pH 5.9), and the flow rate was 0.2 mL min^{-1}. The photocatalytic reaction solution was injected into the UHPLC system within 2 min after light irradiation.

UV-vis absorption measurement during steady-state light irradiation

A CO_2-saturated DMSO–TEOA (5 : 1 v/v) solution containing **RuC2Re** (0.05 mM) and BIH (0.1 M) in an 11 mL necked quartz cubic cell (path length: 1 cm) was irradiated at λ_{ex} = 480 nm (2.6 × 10^{-8} einstein s^{-1}) using a 500 W Xe arc lamp equipped with a bandpass filter. The temporal changes in the UV-vis absorption spectra were measured using a Photal MCPD-9800 photodiode array spectrometer (Otsuka Electronics Co., Ltd.). The sample temperature was maintained at 25 °C ± 0.1 °C using an IWAKI CTS-134A temperature bath.

Quantum chemical calculations

Quantum chemical calculations based on DFT were performed using the *Gaussian 16* package. [7] Geometric optimization and frequency calculations were performed in DMSO (PCM, ε = 46.7) using the ωB97XD function, Lanl2DZ basis set for Re with an added f polarization function, [8] and 6-311G+(d,p) for the other elements. The free energy in the solution was calculated based on the electronic energy and vibrational frequency obtained from the calculations under this condition with a correction of +1.9 kcal/mol for the change in the reference states from 1 atm to 1 M. [9] The calculated vibrational frequencies were scaled by a scaling factor of 0.9618 to account for the anharmonicity in the comparison of the experimental values (Table S1). The stationary points were verified by frequency analysis and the transition states were connected to minima using intrinsic reaction coordinate (IRC) computations.

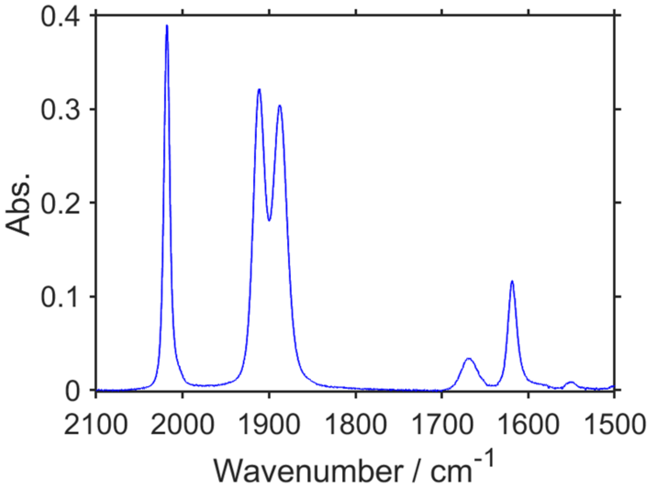

Figure S1. FT-IR spectrum of **RuC2Re** (5 mM) in a CO_2 saturated DMSO-TEOA (5:1 v/v) solution; d = 0.2 mm.

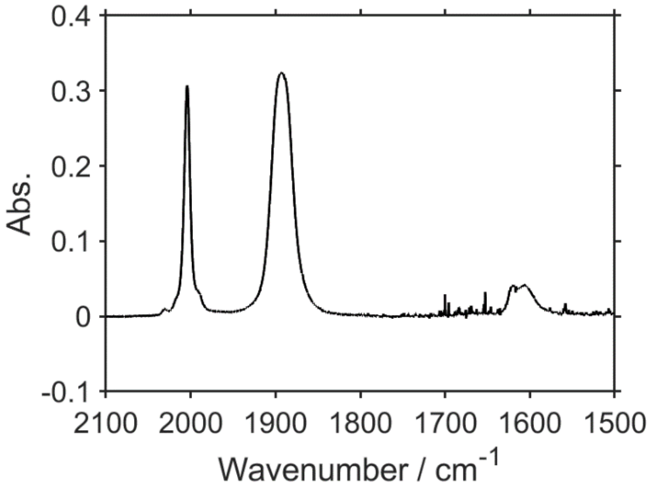

Figure S2. FT-IR spectrum of *fac*-Re(dmb)$(CO)_3$(COOH) (4 mM) immediately after dissolving in an Ar purged DMSO-TEOA (5:1 v/v) solution; d = 0.2 mm.

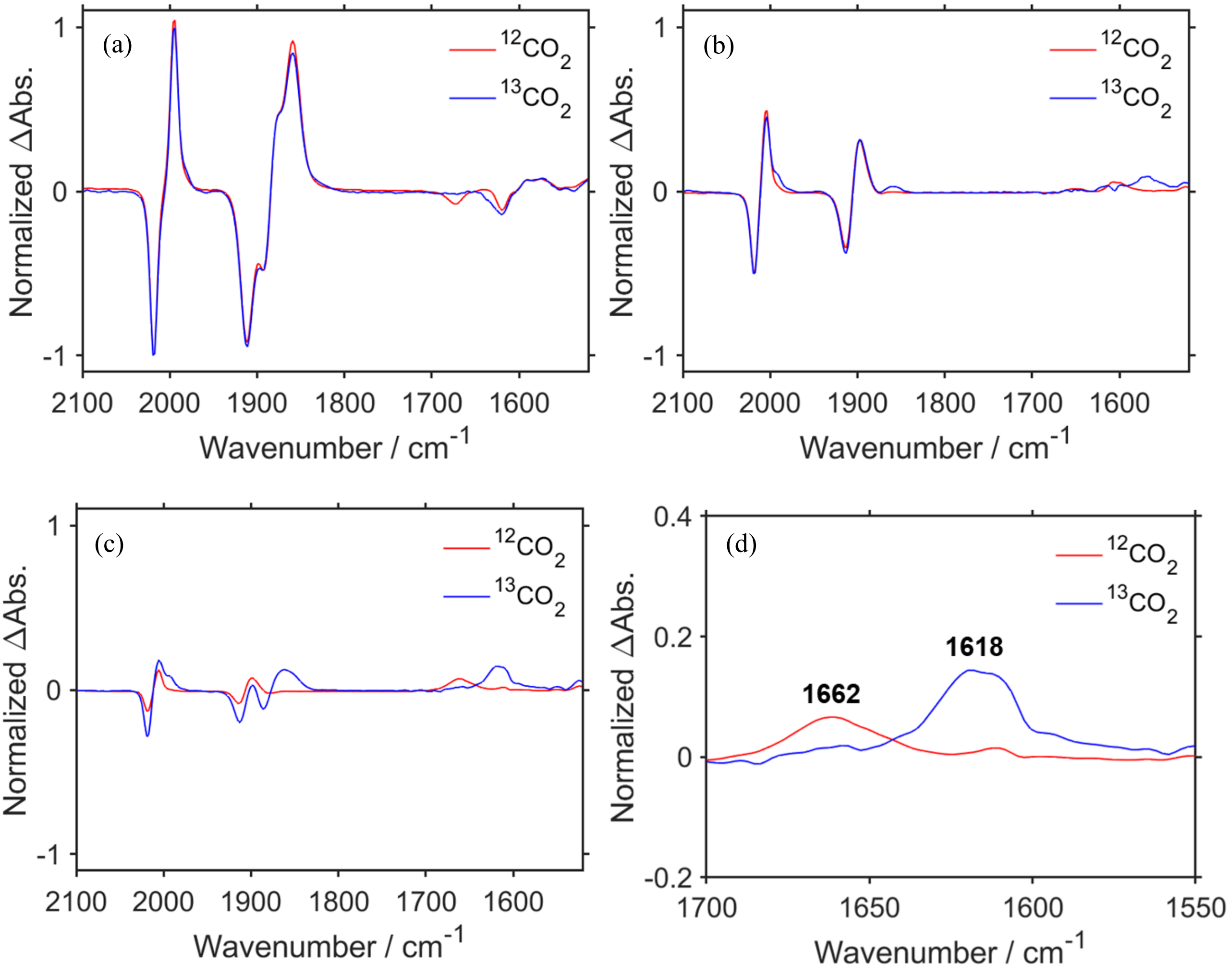

Figure S3. TR-IR spectra of an ordinary CO_2 (red) or $^{13}CO_2$ (blue) saturated DMSO-TEOA (5:1 v/v) solution containing **RuC2Re** (1.0 mM) and BIH (0.1 M) at (a) 21 ms, (b) 3.0 s and (c, d) 5.0 min after the laser flash. These spectra were normalized by absorbance at 2019 cm^{-1} at 21 ms after pulsed excitation. A total of 12 loops of spectra using two samples were averaged for the final data.

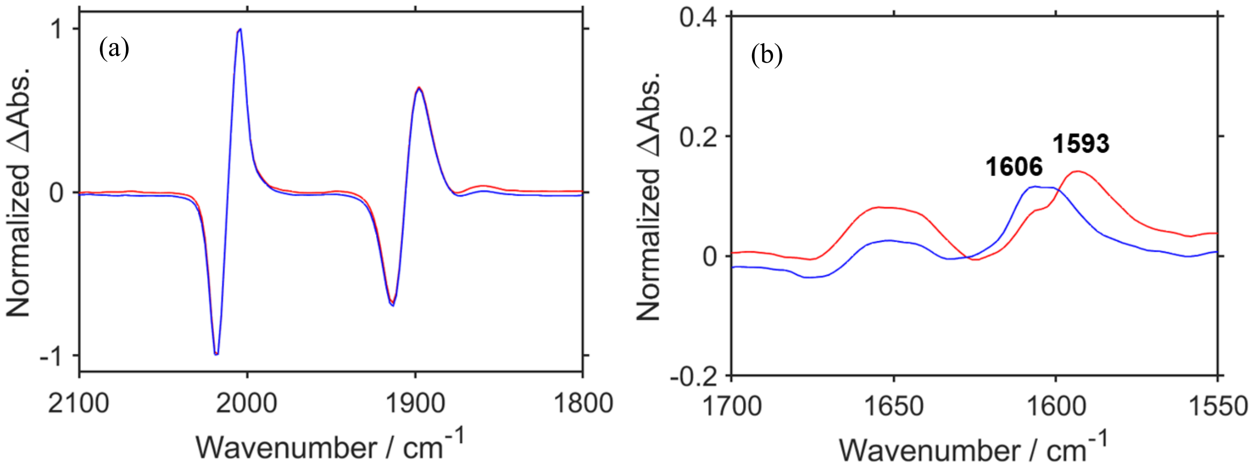

Figure S4. TR-IR spectra of a CO_2 saturated DMSO-TEOA (5:1 v/v) solution containing **RuC2Re** (1.0 mM) and BIH (0.1 M) (blue) and a CO_2 saturated DMSO-TEOA-d_3 (5:1 v/v) containing **RuC2Re** (1.0 mM) and deuterated BIH at the second position of the imidazole ring (red) at 2.0 s after laser flash.

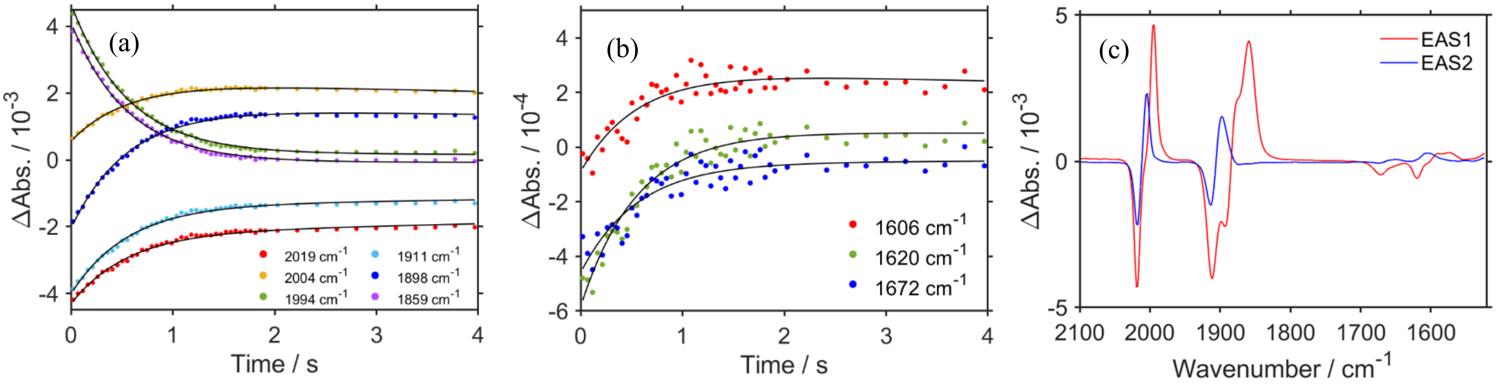

Figure S5. (a,b) Kinetics traces (dots) of TR-IR spectra from 21 ms to 4.0 s at characteristic wavelengths with their fits obtained by global analysis using a two-component global sequential routine (black line). (c) Evolution-associated spectra (EAS) generated by global analysis of TR-IR spectra from 21 ms to 4 s after laser flash using a two component.

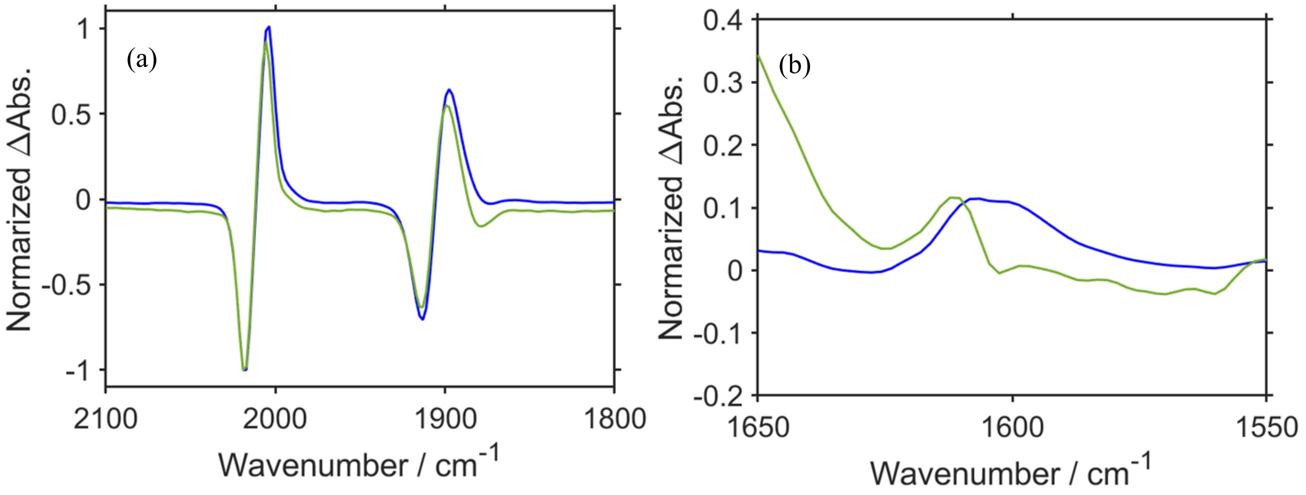

Figure S6. TR-IR spectra at 3.0 s (blue) and 5.0 min (green) in the (a) CO ligand and (b) C=O vibration region after laser flash normalized at 2019 cm^{-1}.

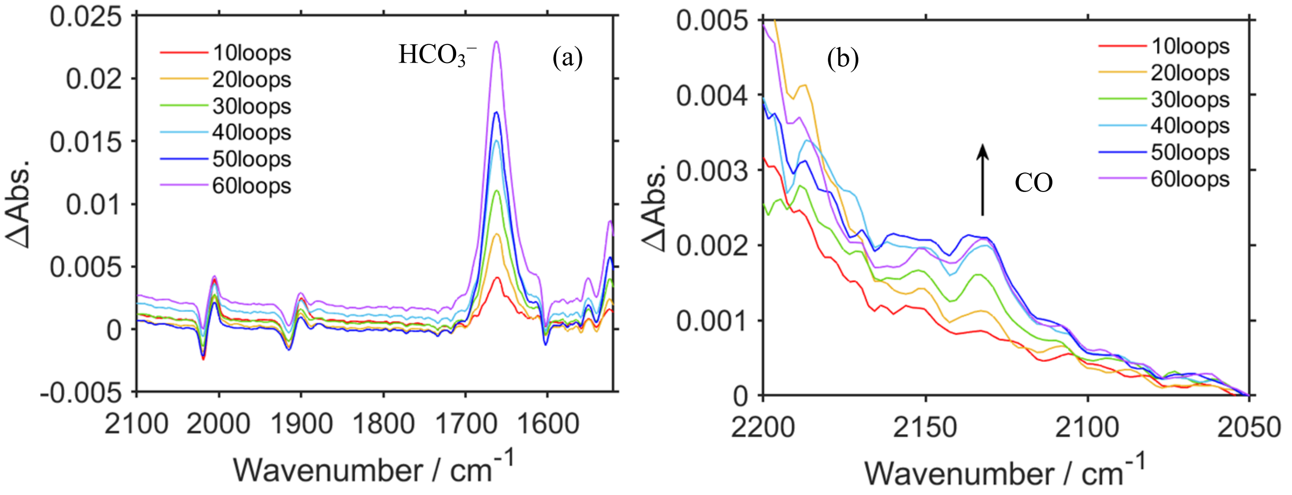

Figure S7. Difference spectra of a CO_2 saturated DMSO-TEOA (5:1 v/v) solution containing **RuC2Re** (1.0 mM) and BIH (0.1 M) between FT-IR spectra per 10 laser flash irradiations and FT-IR spectrum before irradiations. The spectra on the high wavenumber side (b) were aligned to zero absorbance at 2050 cm^{-1}.

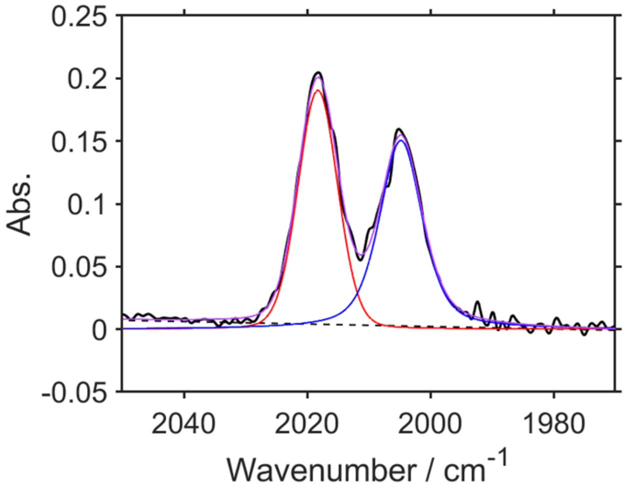

Figure S8. FT-IR spectrum (black line) of a CO_2 saturated DMSO-TEOA (5:1 v/v) solution containing **RuC2Re** (2.0 mM) and BIH (0.1 M) during steady-state light (480 nm) irradiation at 45 s with the fitting curve (purple) and corresponding components; red and blue denote the curves of **RuC2Re** and **RuC2Re(COOH)**, respectively.

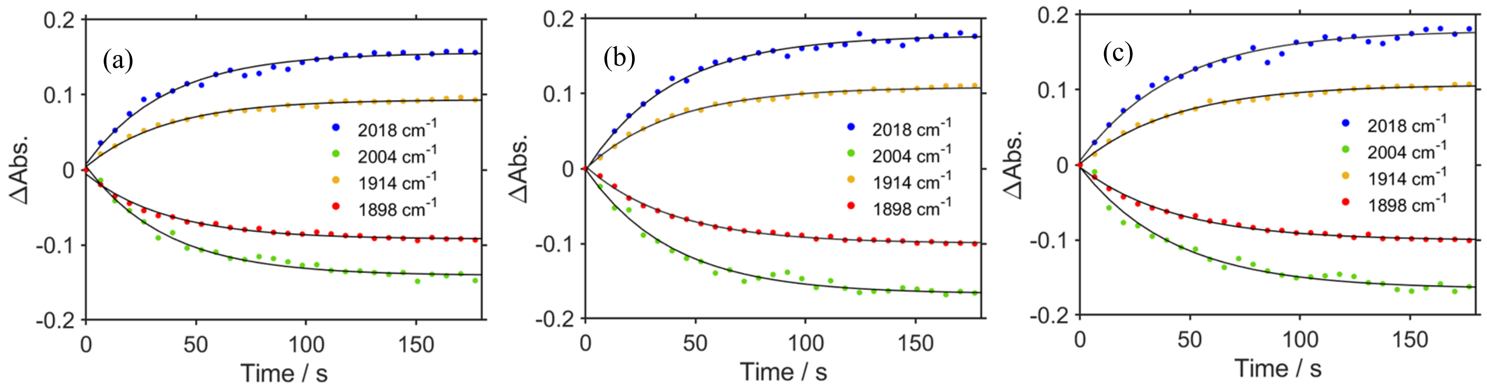

Figure S9. Kinetic traces (dots) of different FT-IR spectra of a CO_2 saturated DMSO-TEOA (5:1 v/v) solution containing **RuC2Re** (2 mM) and BIH (0.1 M) after (a) 1 min, (b) 2 min and (c) 4 min steady-state light irradiation ($\lambda = 480$ nm) with their fits obtained by global analysis in 1800–2100 cm^{-1} using a single exponential function and one constant (black line) at characteristic wavelengths. The time in the figure is the time since light irradiation was stopped.

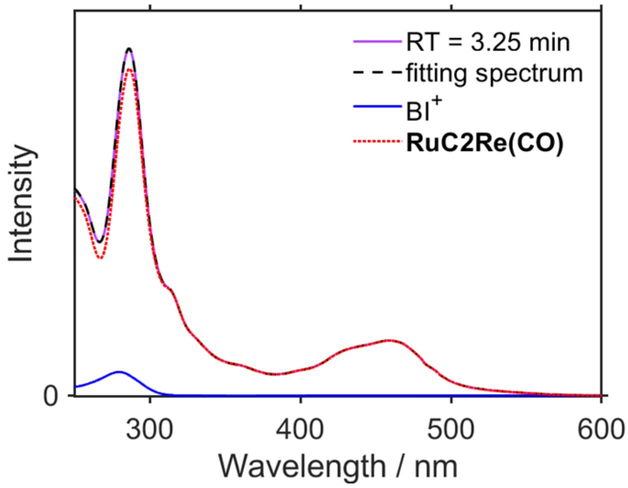

Figure S10. UV-vis absorption spectra of the photocatalytic reaction solution (purple) at 3.25 min, **RuC2Re(CO)** (red) and BI^+ (blue) obtained by UHPLC analysis. The black dash line is the fitting result. Although BI^+ that is a two-electron oxidized and deprotonated species of BIH was also eluted at 3.25 min, the chromatograms shown in Fig. 5a should not be affected by the simultaneous elution of BI^+ because it has no absorption at 460 nm as shown by its spectrum in blue.

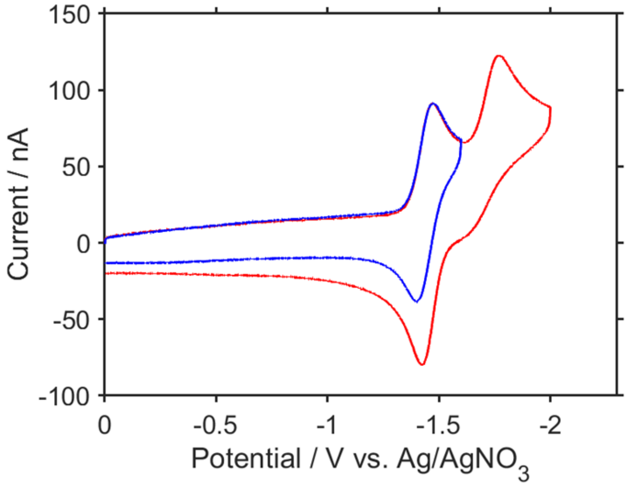

Figure S11. Cyclic voltammogram using ultra microelectrode (d =33 μm) of $[Re(dmb)(CO)_4]^+$ (2 mM) in Ar purged DMSO solution containing Et_4NBF_4. Scan rate: 30 V/s.

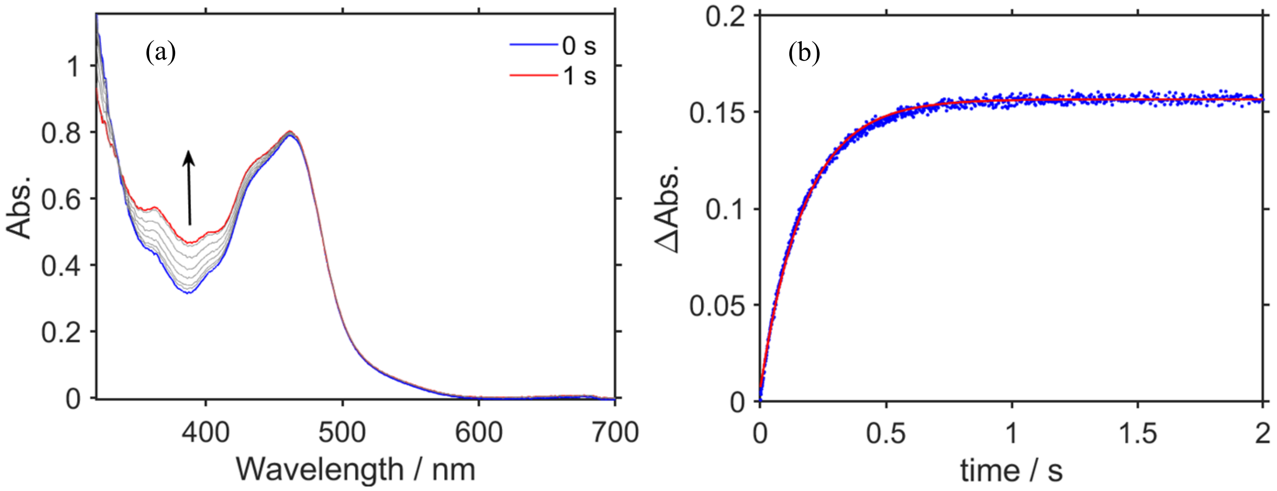

Figure S12. (a) UV-Vis spectra after mixing a DMSO-TEOA (5:2 v/v) solution with the same volume of a DMSO solution containing **RuC2Re(CO)** (0.1 mM). (b) Temporal change (blue) of ΔAbs. at 375 nm with their fitting (red) using a single exponential function and one constant.

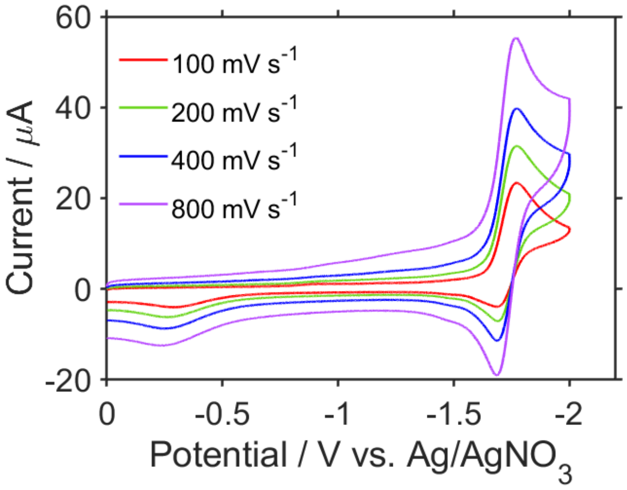

Figure S13. Cyclic Voltammogram of *fac*-Re(dmb)$(CO)_3$(C(=O)$OCH_2CH_2N(CH_2CH_2OH)_2$) (**Re(CO-TEOA)**) in Ar purged DMSO-TEOA (5:1 v/v) solution containing Et_4NBF_4. Scan rate: 100-800 mV s^{-1}.

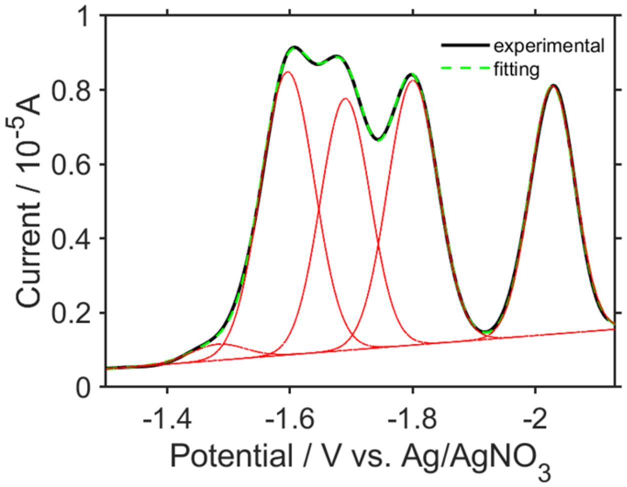

Figure S14. DPV of **RuC2Re(CO-TEOA)** (1 mM) in an Ar purged DMSO-TEOA (5:1 v/v) solution, pulse amplitude = 50 mV.

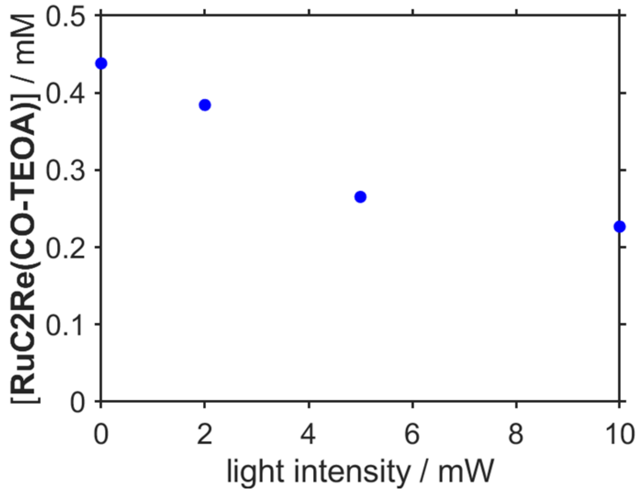

Figure S15. Light intensity dependence of the concentration of **RuC2Re(CO-TEOA)** after 5 min irradiation. An Ar purged DMSO-TEOA (5:1 v/v) solution containing **RuC2Re(CO-TEOA)** (0.5 mM) and BIH (0.1 M) was irradiated with a 530 nm LED light. The x-axis values are the input power of the apparatus. Owing to equipment limitations, the absolute light intensity cannot be calculated.

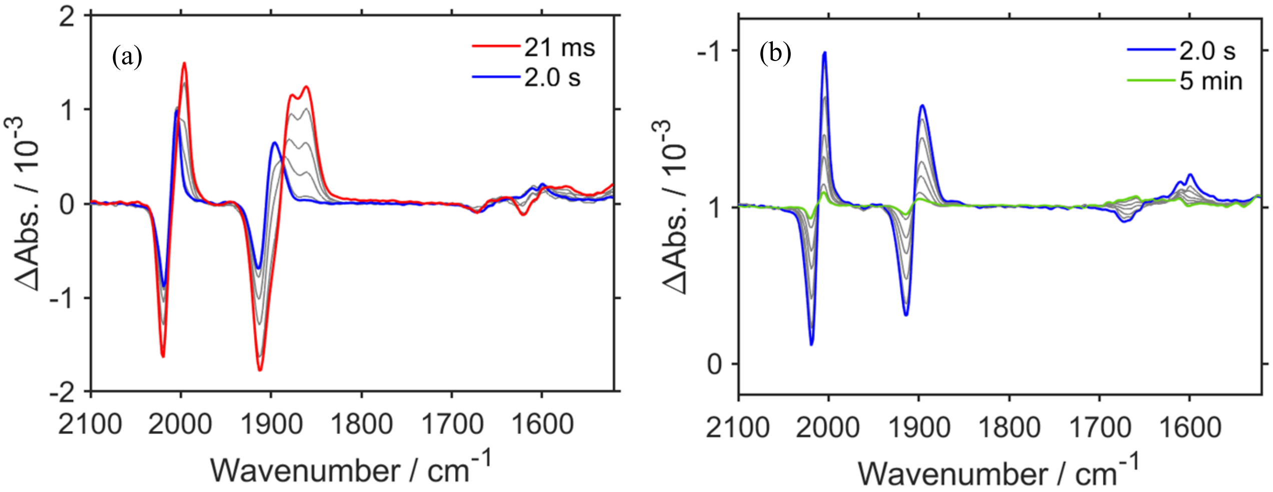

Figure S16. TR-IR spectra of a CO_2 saturated DMSO-TEOA (5:1 v/v) solution containing **RuC2Re** (1.0 mM), BIH (0.1 M) and NH_4PF_6 (20 mM) (a) from 21 ms to 2.0 s and (b) from 2.0 s to 5.0 min after pulsed excitation at 532 nm. A total of 25 loops of spectra were averaged for the final data.

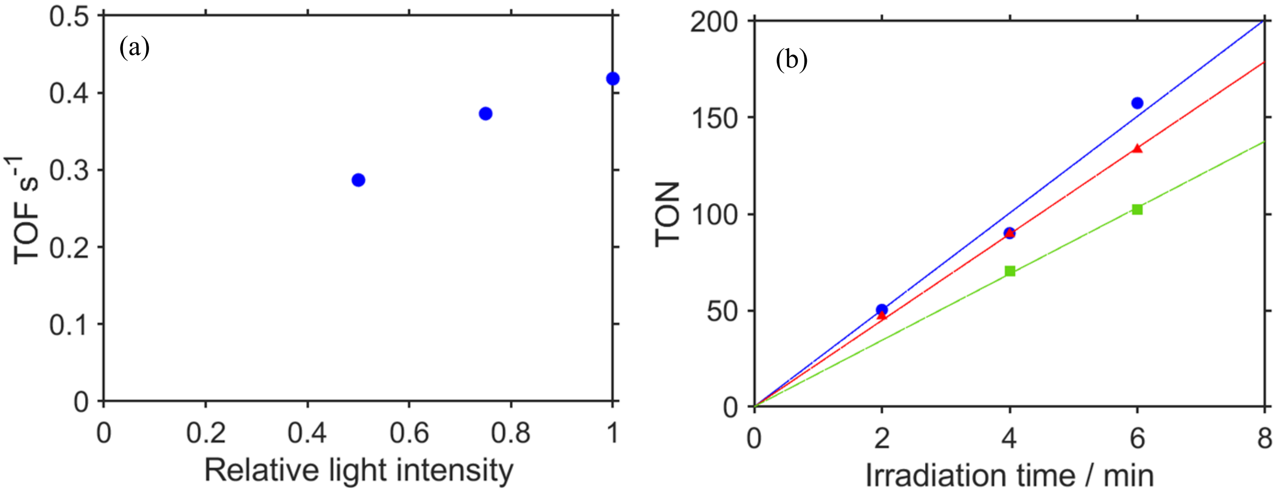

Figure S17. (a) Light intensity dependence of TOF of CO production by **RuC2Re**. (b) Temporal change of TON of CO production. A CO_2 saturated DMSO-TEOA (5:1 v/v) solution containing **RuC2Re** (0.05 mM) and BIH (0.1 M) was irradiated with 440-600 nm (λ_{max} = 470 and 520 nm) LED light.

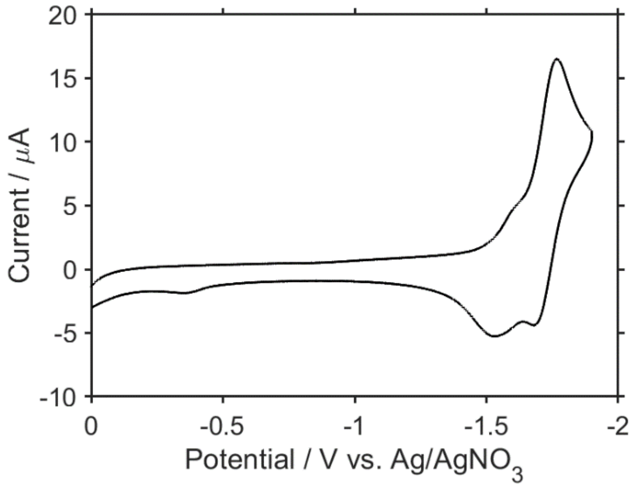

Figure S18. Cyclic Voltammogram of *fac*-Re(dmb)(CO)$_3$(COOH) (**Re(COOH)**) in Ar purged DMSO-TEOA (5:1 v/v) solution containing Et_4NBF_4. Scan rate: 100 mV s^{-1}.

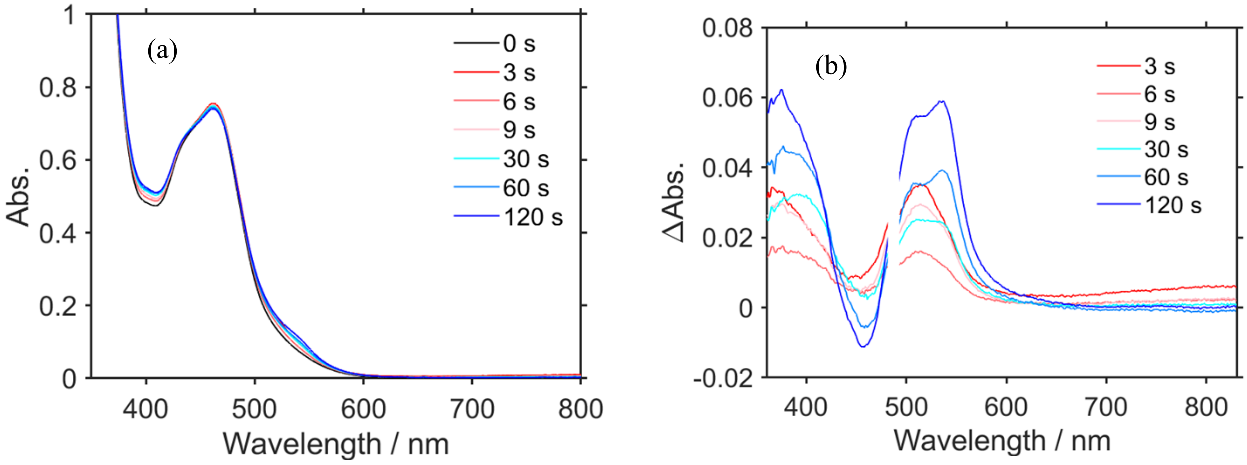

Figure S19. (a) UV-vis absorption spectra and (b) difference absorption spectra of a CO_2 saturated DMSO-TEOA (5:1 v/v) solution containing **RuC2Re** (0.05 mM) and BIH (0.1 M) during irradiation at 480 nm (2.6 × 10^{-8} einstein s^{-1}).

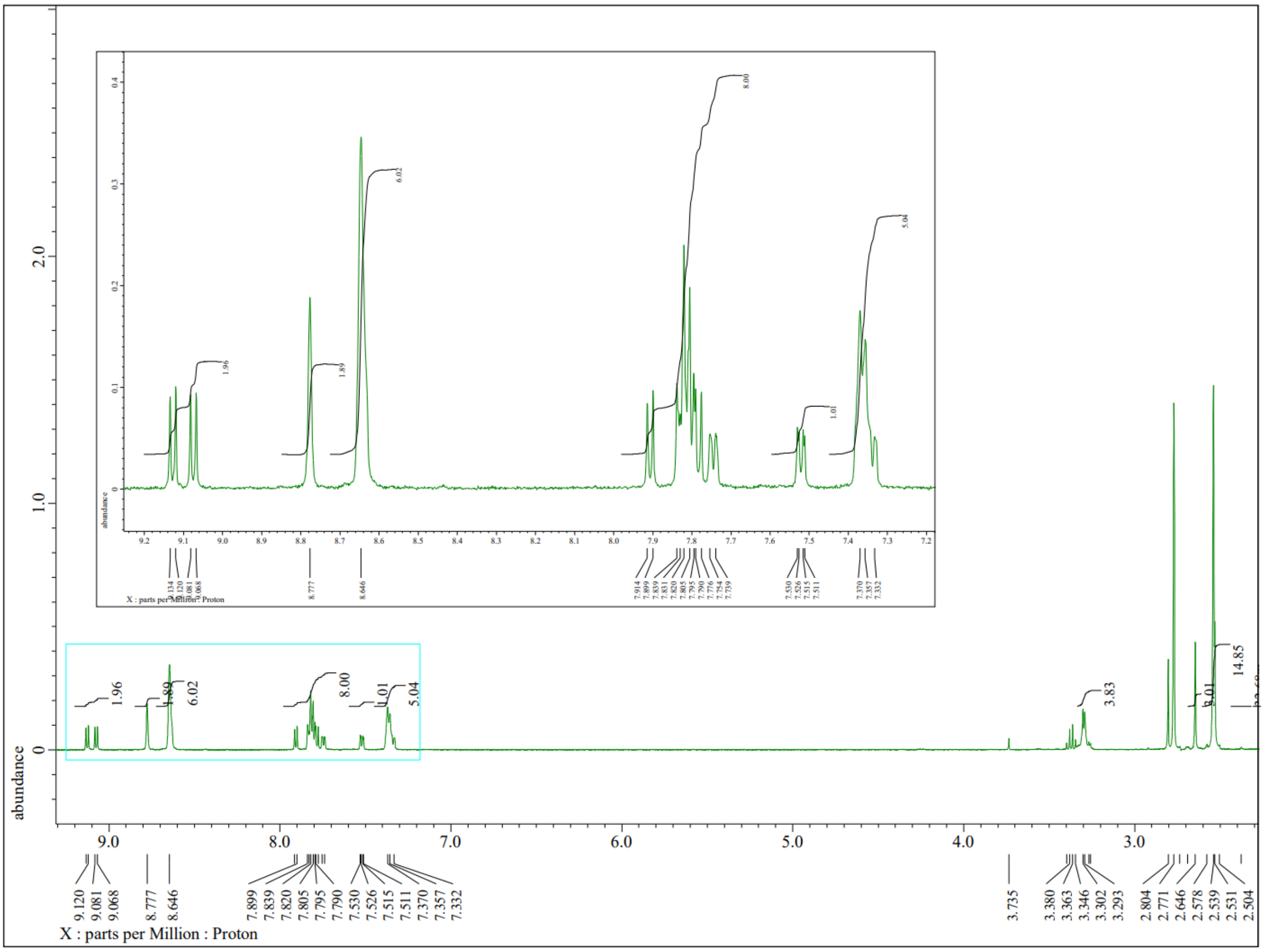

Figure S20. ^{1}H NMR spectrum of **RuC2Re(CO)** in acetone-d_6.

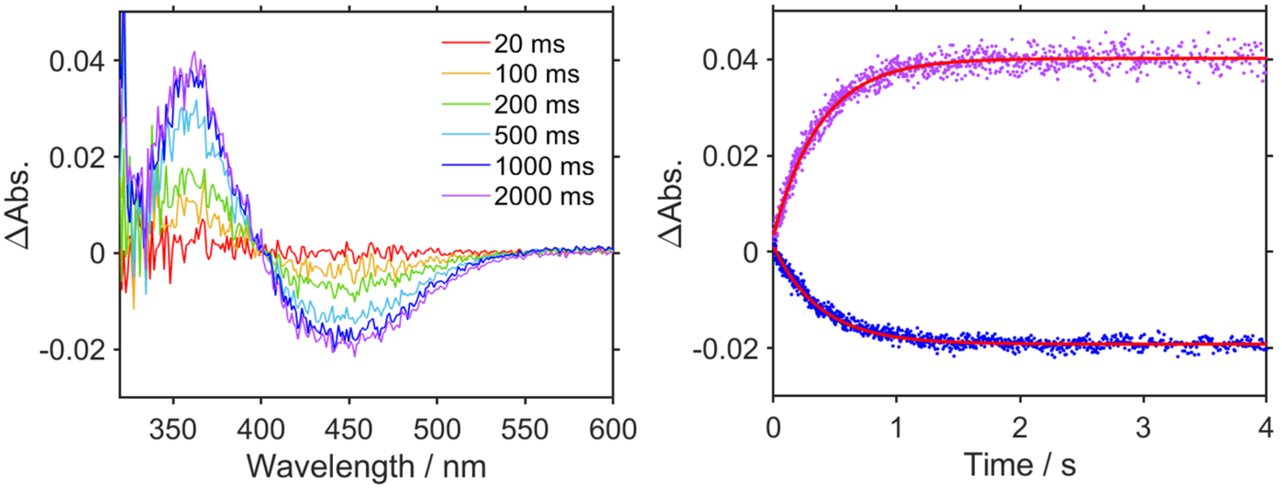

Figure. S21 (a) UV-Vis spectra after mixing an Ar purged DMSO-TEOA (5:1 v/v) solution containing **RuC2Re(DMSO)** and **RuC2Re(TEOA)** complexes (the structures are shown in the reactions below; total concentration of these solvent complex is 0.01 mM) with the same volume of a CO_2 saturated (0.12 M) DMSO-TEOA (5:1 v/v) solution. (b) Temporal change (blue) of ΔAbs. at 360 nm (purple) and 450 nm (blue) with their fitting (red) using a single exponential function and one constant.

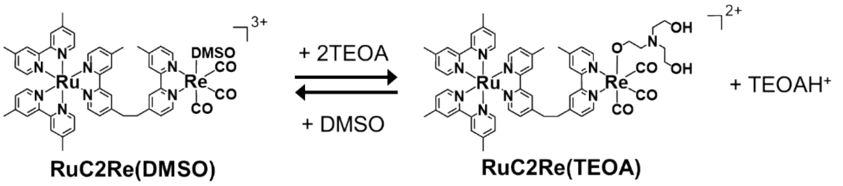

(eq. S1)

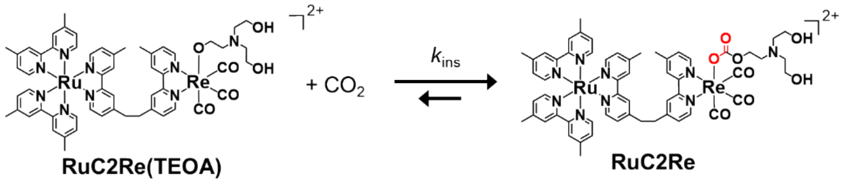

(eq. S2)

After mixing, the UV-Vis absorption was changed owing to the CO_2 capture reaction (forward reaction of eq. S2) with an isosbestic point at 400 nm. Although the complex solution before mixing also contains a DMSO complex **RuC2Re(DMSO)**, the ligand exchange reaction (eq. S1) occurred for a time scale of a few hours and could be ignored on the time scale of this experiment. The CO_2 concentration change could be ignored because of the higher concentration of CO_2 (0.060 M; half of the saturation concentration) after mixing compared with the concentration of the complex. The CO_2 saturation concentration in the DMSO-TEOA (5:1 v/v) mixed solution was determined by a previously reported titration method. [10] The CO_2 dissociation reaction (backward reaction of eq. S2) could also be ignored because of the higher equilibrium constant of eq. S2. [10-12] Thus, the UV-Vis spectra shown in Figure S21a could be analyzed using a simple pseudo-first-order kinetics model (the apparent rate constant is $0.060k_{ins}$) and the bimolecular rate constant k_{ins} was determined to be 44 M^{-1} s^{-1}.

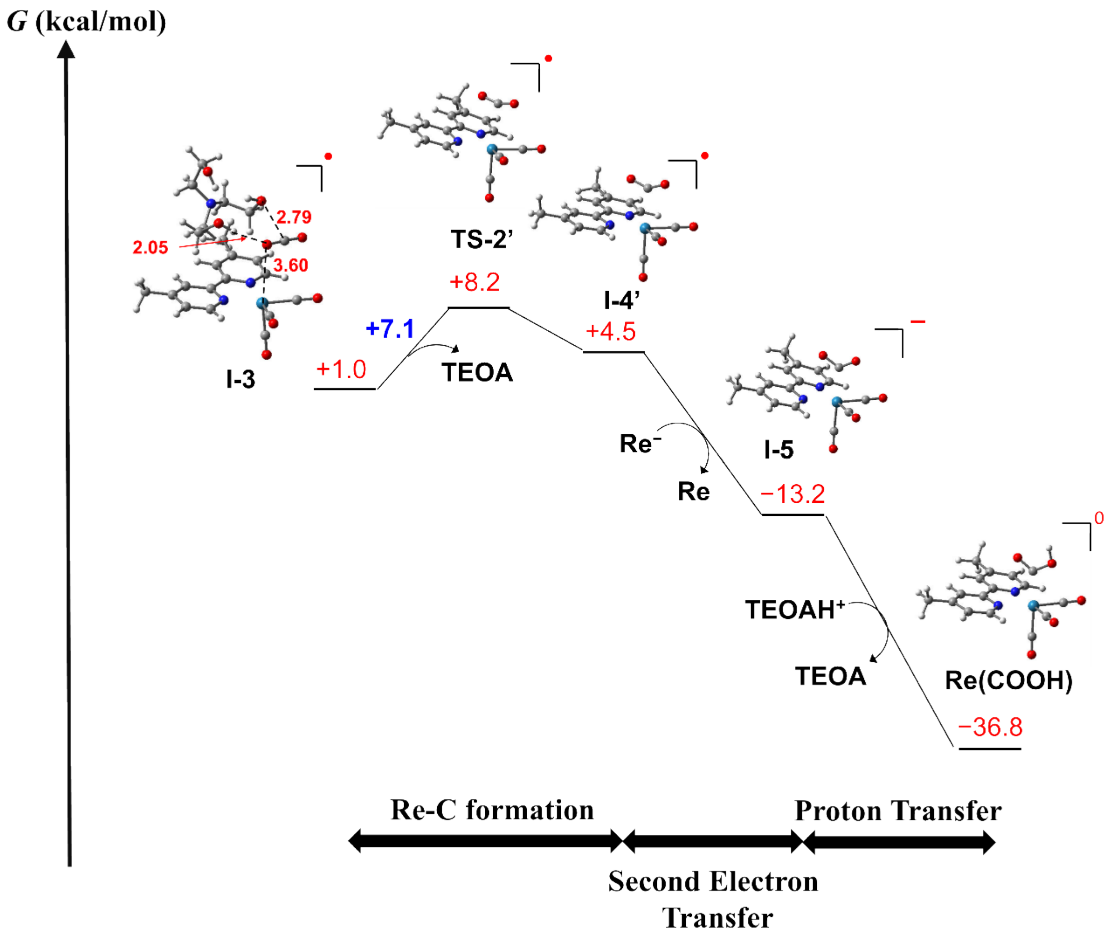

Scheme S1. Computed free energy profile for the conversion reaction from one-electron reduced species of *fac*-Re(dmb)$(CO)_3(OC(=O)OCH_2CH_2N(CH_2CH_2OH)_2)$ (**Re⁻**) to *fac*-Re(dmb)$(CO)_3$(COOH) (**Re(COOH)**). Re-C bond formation occurs before the second electron reduction.

Table S1 IR stretching frequencies (cm^{-1}) calculated by DFT for Re mononuclear model complexes. (frequency scaling factor = 0.9618)

Complex (R = CH_2CH_2OH)	CO ligands	C=O	Diimine
[Re(dmb)$(CO)_3(O(C{=}O)OC_2H_4NR_2)$]	2034, 1895, 1880	1653	1635, 1626
[Re(dmb)$(CO)_3(O(^{13}C{=}O)OC_2H_4NR_2)$]	2034, 1895, 1880	1608	1635, 1626
[Re(dmb$^{\bullet-}$)$(CO)_3(O(C{=}O)OC_2H_4NR_2)]^-$	2012, 1862, 1851	1651	1603, 1601
Re(dmb)$(CO)_3$(COOH)	2019, 1885, 1874	1610	1635, 1627
Re(dmb)$(CO)_3$(COOD)	2018, 1885, 1874	1601	1635, 1627
Re(dmb)$(CO)_3(^{13}COOH)$	2018, 1885, 1874	1573	1635, 1627

Optimized coordinates calculated by DFT method

Re⁻ (Charge: −1, Doublet)

Symbol	X	Y	Z
C	-0.64981	-1.80122	-1.96729
C	-3.13686	-2.04551	-1.05722
C	-1.07263	-2.76831	0.457008
O	-0.09627	-2.19127	-2.90527
O	-4.08315	-2.59518	-1.44184
O	-0.77761	-3.74811	0.996386
N	-2.59117	-0.18535	1.222284
C	-2.79813	-0.72602	2.437784
C	-3.01887	1.109318	0.994043
C	-3.40612	-0.0687	3.475816
H	-2.44966	-1.74448	2.565388
C	-3.65405	1.829248	2.044761
C	-2.78967	1.62818	-0.30524
C	-3.85346	1.266563	3.275934
H	-3.53737	-0.56744	4.428203
H	-3.98752	2.844361	1.866819
C	-3.16196	2.936578	-0.72402
N	-2.16496	0.782182	-1.20255
C	-2.91598	3.375266	-1.99655
H	-3.64966	3.600542	-0.02075
C	-1.93394	1.227103	-2.45194
C	-2.27598	2.478233	-2.8958
H	-2.0565	2.768199	-3.91598
H	-1.44651	0.525381	-3.11899
Re	-1.59191	-1.16469	-0.43629
O	0.076283	0.03271	0.328607
C	1.293708	-0.31539	0.423608
O	1.819792	-1.37898	0.112018
O	2.042276	0.699643	0.965324
C	3.444728	0.482599	1.080188
H	3.785806	1.147163	1.876185
H	3.639115	-0.55026	1.374704
C	4.164552	0.824242	-0.21927
H	3.68801	0.293805	-1.05765

H	4.051991	1.895629	-0.40502
N	5.583111	0.511477	-0.11724
C	5.897755	-0.90411	-0.30111
H	4.990655	-1.49145	-0.13127
H	6.219137	-1.10819	-1.33334
C	6.95071	-1.40727	0.679043
H	6.580596	-1.26013	1.702184
H	7.097713	-2.4792	0.524521
C	6.449002	1.425907	-0.85992
H	5.947726	1.847814	-1.7418
H	7.319626	0.872966	-1.21721
C	6.922349	2.544431	0.063304
H	7.726444	3.117022	-0.40092
H	6.098597	3.2313	0.293024
O	7.437254	1.989472	1.270455
H	6.708619	1.456899	1.616666
O	8.228083	-0.80448	0.52501
H	8.166055	0.120275	0.800012
C	-4.52148	2.010701	4.396253
H	-4.8091	3.018846	4.092892
H	-5.41916	1.481213	4.729658
H	-3.85408	2.087367	5.259982
C	-3.29955	4.752793	-2.4553
H	-3.98506	4.699116	-3.30648
H	-3.78335	5.32072	-1.65873
H	-2.41711	5.30788	-2.78781

I1 (Charge: 0, Doublet)

Symbol	X	Y	Z
C	1.015163	-2.91973	1.538075
C	3.486344	-2.74256	0.56969
C	1.444479	-3.23176	-1.05492
O	0.517046	-3.56757	2.355375
O	4.479762	-3.29386	0.796635
O	1.219101	-4.07264	-1.81467
N	2.765682	-0.43772	-1.17343
C	2.984705	-0.65367	-2.48374

C	3.198961	0.751603	-0.6186
C	3.625963	0.23211	-3.31108
H	2.621113	-1.59745	-2.87265
C	3.874522	1.700369	-1.43928
C	2.955261	0.926404	0.766557
C	4.106454	1.45506	-2.76834
H	3.766339	-0.00913	-4.35699
H	4.2386	2.620966	-0.99939
C	3.293637	2.099306	1.497585
N	2.34982	-0.12932	1.420997
C	3.066695	2.188825	2.84419
H	3.740397	2.93888	0.979284
C	2.144394	-0.03073	2.749087
C	2.476201	1.070803	3.493844
H	2.276187	1.082758	4.558003
H	1.680902	-0.88988	3.219093
Re	1.866216	-1.85705	0.201047
O	0.056984	-0.6602	-0.23269
C	-1.09279	-1.15802	-0.44061
O	-1.47152	-2.31517	-0.40427
O	-1.98724	-0.14485	-0.75381
C	-3.36685	-0.49161	-0.89357
H	-3.80267	0.255096	-1.55902
H	-3.44537	-1.47118	-1.36534
C	-4.06834	-0.45942	0.45706
H	-3.55364	-1.13004	1.16214
H	-3.99034	0.553591	0.861064
N	-5.47616	-0.7893	0.311064
C	-5.75446	-2.21966	0.193332
H	-4.83707	-2.73673	-0.10327
H	-6.05932	-2.6449	1.160609
C	-6.80754	-2.52611	-0.86495
H	-6.45228	-2.15622	-1.83562
H	-6.93156	-3.60921	-0.94101
C	-6.34907	-0.0726	1.240204
H	-5.84698	0.151846	2.191337
H	-7.20882	-0.70413	1.469819

C	-6.84401	1.213807	0.586949
H	-7.64615	1.661952	1.174736
H	-6.03036	1.945446	0.503724
O	-7.37179	0.929595	-0.70513
H	-6.64531	0.49697	-1.17256
O	-8.09388	-1.99749	-0.57548
H	-8.05591	-1.03483	-0.6518
C	4.842364	2.428734	-3.64317
H	5.111101	3.335018	-3.09818
H	5.758537	1.973901	-4.03094
H	4.232199	2.709584	-4.50665
C	3.406828	3.421248	3.629602
H	4.11125	3.183961	4.432086
H	3.849701	4.191337	2.996137
H	2.510879	3.836147	4.102162
H	-1.18589	1.389765	-0.28828
N	-0.95867	2.325115	0.094878
C	0.166654	2.891153	-0.72501
H	-0.00377	3.958057	-0.85426
H	1.091676	2.738652	-0.16848
C	-2.23726	3.110183	0.022344
H	-2.9379	2.600976	0.684171
H	-2.04628	4.098676	0.434805
C	-0.55128	2.040817	1.5144
H	0.260532	1.317739	1.443841
H	-1.3997	1.552562	1.996523
C	0.277116	2.203376	-2.08461
H	-0.67718	2.239936	-2.60926
H	0.559731	1.154716	-1.94448
C	-0.11003	3.24067	2.320863
H	0.343647	2.841576	3.233623
H	0.662631	3.808468	1.78986
C	-2.84501	3.211305	-1.3598
H	-2.80391	2.243789	-1.87436
H	-3.90059	3.469393	-1.22497
O	-2.17141	4.212964	-2.09951
H	-2.56187	4.265894	-2.97409

O	1.208302	2.894872	-2.88975
H	2.09441	2.628485	-2.61897
O	-1.22634	4.05899	2.626961
H	-0.92478	4.798267	3.158466

TS1 (Charge: 0, Doublet)

Symbol	X	Y	Z
C	1.535015	-3.04005	1.300085
C	4.00063	-2.36094	0.630108
C	2.288825	-3.04259	-1.26402
O	1.076297	-3.82567	2.009109
O	5.047455	-2.74154	0.937257
O	2.288856	-3.83023	-2.10644
N	2.982781	-0.06127	-1.03985
C	3.37365	-0.11788	-2.33281
C	3.075022	1.149493	-0.37982
C	3.86687	0.951109	-3.02816
H	3.279516	-1.0846	-2.81209
C	3.59209	2.284277	-1.06286
C	2.661053	1.152607	0.976389
C	3.998285	2.205228	-2.36945
H	4.158906	0.831519	-4.06371
H	3.688428	3.22419	-0.5331
C	2.661553	2.301301	1.810792
N	2.244716	-0.06159	1.484749
C	2.283127	2.226841	3.12339
H	2.968651	3.255241	1.400484
C	1.874668	-0.1196	2.784579
C	1.878732	0.959002	3.624076
H	1.565791	0.839549	4.653872
H	1.563164	-1.09246	3.144094
Re	2.31024	-1.73309	0.134118
O	0.206088	-0.84851	-0.4828
C	-0.72172	-1.52763	-0.79616
O	-1.27494	-2.53331	-1.00075
O	-2.01937	-0.16499	-1.22185
C	-3.38867	-0.54269	-1.1984

H	-3.97945	0.198295	-1.74572
H	-3.45969	-1.48729	-1.74095
C	-3.92885	-0.69173	0.218784
H	-3.2699	-1.35312	0.80384
H	-3.90804	0.286754	0.70492
N	-5.30329	-1.16087	0.199046
C	-5.45194	-2.60411	0.025591
H	-4.52699	-3.00722	-0.39773
H	-5.60339	-3.10604	0.992454
C	-6.58066	-2.96833	-0.93188
H	-6.37518	-2.51497	-1.91034
H	-6.60177	-4.05341	-1.06011
C	-6.14498	-0.57828	1.243134
H	-5.57363	-0.33701	2.149964
H	-6.90706	-1.30658	1.525353
C	-6.83359	0.671707	0.70532
H	-7.60586	1.015259	1.394715
H	-6.11007	1.48549	0.568866
O	-7.47081	0.381017	-0.53498
H	-6.76025	0.060206	-1.10504
O	-7.8733	-2.59576	-0.47632
H	-7.94334	-1.63209	-0.50394
C	4.564497	3.385412	-3.10373
H	4.57749	4.279013	-2.47789
H	5.587308	3.175933	-3.43004
H	3.97697	3.597812	-4.00178
C	2.273177	3.428416	4.021069
H	2.932411	3.272277	4.879894
H	2.598415	4.326103	3.493145
H	1.267733	3.604855	4.415702
H	-1.83739	0.72962	-0.75769
N	-1.51485	2.150911	0.000381
C	-0.35888	2.829798	-0.62368
H	-0.52702	3.909599	-0.67124
H	0.527768	2.661366	-0.00664
C	-2.75699	2.943209	-0.06601
H	-3.51135	2.406903	0.51425

H	-2.6292	3.918041	0.417892
C	-1.21825	1.692773	1.370769
H	-0.32444	1.066579	1.310452
H	-2.03743	1.047506	1.70318
C	-0.07237	2.315314	-2.03305
H	-0.97873	2.350577	-2.63762
H	0.272431	1.276047	-1.98875
C	-0.98683	2.75454	2.43366
H	-0.51039	2.264871	3.289692
H	-0.2988	3.525298	2.06392
C	-3.31103	3.164567	-1.46192
H	-3.21875	2.252119	-2.06305
H	-4.37763	3.398209	-1.36746
O	-2.6275	4.243143	-2.08645
H	-2.97943	4.35474	-2.97139
O	0.879042	3.13528	-2.69226
H	1.743589	2.96132	-2.30501
O	-2.22773	3.329707	2.825371
H	-2.05428	3.987067	3.501502

I2 (Charge: 0, Doublet)

Symbol	X	Y	Z
C	2.025616	-3.03635	1.278657
C	4.378913	-2.054	0.624236
C	2.790075	-2.91943	-1.28693
O	1.664003	-3.87813	1.978461
O	5.464032	-2.29961	0.935798
O	2.892177	-3.69023	-2.13795
N	3.036187	0.118493	-1.04159
C	3.43391	0.121998	-2.33785
C	2.969064	1.328355	-0.37881
C	3.787433	1.249629	-3.02125
H	3.465657	-0.8448	-2.82427
C	3.343598	2.522535	-1.04847
C	2.552822	1.266569	0.977198
C	3.761555	2.505666	-2.35404
H	4.094536	1.175678	-4.05675

H	3.321099	3.462069	-0.50982
C	2.414931	2.394772	1.824015
N	2.294548	0.002627	1.466242
C	2.049594	2.258163	3.135808
H	2.608057	3.383265	1.426278
C	1.92569	-0.1162	2.765925
C	1.796684	0.944141	3.615608
H	1.496989	0.774405	4.642332
H	1.734947	-1.1238	3.112842
Re	2.634566	-1.63107	0.12507
O	0.257758	-1.08489	-0.55255
C	-0.50818	-1.91954	-0.8358
O	-1.17837	-2.82066	-1.08631
O	-2.25489	-0.24226	-1.46142
C	-3.60312	-0.6501	-1.37113
H	-4.27799	0.099939	-1.80543
H	-3.69746	-1.55685	-1.97473
C	-4.03687	-0.92317	0.06569
H	-3.3346	-1.62659	0.541566
H	-3.97478	0.013746	0.625624
N	-5.41149	-1.39287	0.127774
C	-5.58895	-2.80798	-0.18863
H	-4.70224	-3.16862	-0.7184
H	-5.6784	-3.41097	0.726749
C	-6.78923	-3.05752	-1.09416
H	-6.64424	-2.50908	-2.03407
H	-6.84542	-4.12328	-1.32954
C	-6.13834	-0.93601	1.310699
H	-5.48145	-0.82306	2.184673
H	-6.89368	-1.68091	1.566722
C	-6.83674	0.384553	1.000461
H	-7.54619	0.64186	1.787992
H	-6.10732	1.200517	0.918353
O	-7.57065	0.270545	-0.21437
H	-6.91329	-0.01013	-0.86496
O	-8.03708	-2.71306	-0.50889
H	-8.07829	-1.75228	-0.41478

C	4.181727	3.751528	-3.07732
H	4.081604	4.635657	-2.446
H	5.224097	3.671446	-3.39886
H	3.576893	3.896029	-3.97729
C	1.905019	3.435557	4.053188
H	2.603213	3.356132	4.891568
H	2.094665	4.375364	3.532601
H	0.896776	3.471565	4.476776
H	-2.11065	0.593496	-0.95505
N	-1.75443	2.097452	0.019087
C	-0.6476	2.859367	-0.5846
H	-0.88494	3.92671	-0.62489
H	0.250436	2.748148	0.031265
C	-3.03229	2.821524	0.027461
H	-3.74209	2.216959	0.597422
H	-2.9508	3.781378	0.554171
C	-1.40804	1.529709	1.328128
H	-0.47094	0.979015	1.20455
H	-2.1709	0.794228	1.603029
C	-0.33653	2.370682	-1.99717
H	-1.24669	2.375679	-2.59774
H	0.041176	1.34147	-1.96002
C	-1.24035	2.495835	2.491609
H	-0.71648	1.965946	3.294644
H	-0.61976	3.350274	2.192113
C	-3.6369	3.073034	-1.34282
H	-3.52404	2.18573	-1.97668
H	-4.70881	3.262084	-1.21131
O	-3.01502	4.199736	-1.94986
H	-3.38691	4.315741	-2.82589
O	0.584165	3.224676	-2.65787
H	1.453541	3.084714	-2.26879
O	-2.51607	2.934106	2.945904
H	-2.38293	3.539619	3.677288

I3 (Charge: 0, Doublet)

Symbol	X	Y	Z

C	2.96768	-1.90046	-0.54731
C	3.877611	0.309701	0.501235
C	3.032358	0.359651	-1.97339
O	3.412935	-2.95598	-0.68634
O	4.872451	0.60082	1.017189
O	3.49653	0.663907	-2.98517
N	1.129748	1.649908	0.03055
C	1.219321	2.779959	-0.72221
C	0.253411	1.653009	1.091217
C	0.479579	3.901349	-0.4884
H	1.923267	2.745321	-1.54346
C	-0.5141	2.802945	1.379523
C	0.162415	0.423903	1.810043
C	-0.4287	3.928422	0.60201
H	0.594749	4.761028	-1.13699
H	-1.19404	2.789806	2.221804
C	-0.72504	0.193074	2.883879
N	0.98094	-0.58683	1.354321
C	-0.78739	-1.02668	3.505907
H	-1.38142	0.990008	3.209943
C	0.930789	-1.78435	2.00207
C	0.092391	-2.04159	3.044684
H	0.096973	-3.02284	3.502874
H	1.600635	-2.5491	1.63138
Re	2.257031	-0.1368	-0.29271
O	-0.48879	-1.30005	-2.3095
C	-0.23632	-2.38881	-1.99933
O	0.06368	-3.46399	-1.70772
O	-2.75269	0.669467	-2.53189
C	-2.81283	1.55139	-1.41711
H	-1.95266	2.230966	-1.40796
H	-3.70949	2.15452	-1.57211
C	-2.86326	0.814787	-0.08391
H	-1.88828	0.35034	0.079873
H	-2.99502	1.556544	0.721311
N	-3.8697	-0.23379	-0.04501
C	-3.6177	-1.24038	0.981931

H	-3.37923	-0.79455	1.959825
H	-4.52822	-1.8337	1.097847
C	-2.49414	-2.17125	0.540432
H	-1.52288	-1.66302	0.593406
H	-2.443	-3.03933	1.200522
C	-5.25794	0.230517	-0.08575
H	-5.28355	1.305855	-0.28162
H	-5.7567	0.074909	0.879277
C	-6.02339	-0.48816	-1.18538
H	-5.96821	-1.57271	-1.0144
H	-7.07717	-0.19884	-1.16214
O	-5.53159	-0.1638	-2.47461
H	-4.56282	-0.12667	-2.44215
O	-2.73822	-2.62376	-0.78251
H	-3.20639	-1.88533	-1.2008
C	-1.26313	5.146927	0.862125
H	-1.88577	5.024008	1.749344
H	-0.6299	6.027774	1.000412
H	-1.91589	5.34919	0.007583
C	-1.75497	-1.31154	4.615221
H	-1.22974	-1.66488	5.506997
H	-2.33297	-0.42507	4.880402
H	-2.45195	-2.10091	4.316576
H	-1.97886	0.104059	-2.44383

TS2’ (Charge: 0, Doublet)

C	2.492746	1.326702	0.13198
C	1.612967	0.000019	-2.05907
C	2.493458	-1.3255	0.1323
O	3.285916	2.134644	0.366121
O	1.938695	-0.00005	-3.17053
O	3.287059	-2.13294	0.366681
N	-0.55347	-1.31186	-0.22614
C	-0.48665	-2.65816	-0.2497
C	-1.77396	-0.73567	-0.15887
C	-1.59899	-3.46287	-0.19663
H	0.504445	-3.08625	-0.3131

C	-2.93544	-1.50081	-0.10176
C	-1.77425	0.734889	-0.15899
C	-2.87063	-2.88541	-0.11619
H	-1.47586	-4.53869	-0.21773
H	-3.90131	-1.01744	-0.04637
C	-2.93601	1.499581	-0.10159
N	-0.55402	1.311556	-0.22675
C	-2.87176	2.884202	-0.11625
H	-3.90167	1.015837	-0.04573
C	-0.48774	2.657861	-0.25062
C	-1.60039	3.462148	-0.19733
H	-1.47769	4.538012	-0.21872
H	0.503153	3.086343	-0.31445
Re	1.170131	0.0002	-0.22392
O	-0.51453	0.003431	2.630002
C	0.665827	0.000415	2.652021
O	1.739651	-0.00251	3.139444
C	-4.1028	-3.73678	-0.05037
H	-5.00831	-3.13004	-0.03464
H	-4.1472	-4.40897	-0.91079
H	-4.08517	-4.35815	0.848767
C	-4.10422	3.735093	-0.04959
H	-4.14704	4.410851	-0.90726
H	-5.00964	3.12813	-0.03804
H	-4.08847	4.352689	0.852197

I-4' (Charge: 0, Doublet)

C	2.530982	1.324455	0.02998
C	1.430226	-0.00013	-2.03918
C	2.530945	-1.32448	0.030183
O	3.349481	2.125979	0.151895
O	1.618122	-0.00022	-3.17755
O	3.349414	-2.12601	0.152256
N	-0.57039	-1.32173	-0.17795
C	-0.50202	-2.66054	-0.19778
C	-1.78582	-0.7399	-0.11959
C	-1.61963	-3.46713	-0.15434

H	0.488558	-3.09139	-0.25199
C	-2.94668	-1.49729	-0.07445
C	-1.78581	0.739931	-0.1196
C	-2.88467	-2.88727	-0.08604
H	-1.4978	-4.54275	-0.17516
H	-3.91277	-1.0134	-0.03092
C	-2.94666	1.497332	-0.07449
N	-0.57037	1.321735	-0.17795
C	-2.88463	2.887313	-0.08608
H	-3.91276	1.013461	-0.03099
C	-0.50198	2.660548	-0.19777
C	-1.61958	3.467156	-0.15435
H	-1.49773	4.542773	-0.17515
H	0.488606	3.091386	-0.25196
Re	1.168273	-5E-06	-0.16149
O	-0.33984	-0.00007	2.558107
C	0.853746	0.00006	2.338334
O	1.91851	0.000211	2.918121
C	-4.12596	-3.72346	-0.00447
H	-4.99231	-3.18776	-0.39414
H	-4.00662	-4.65752	-0.55519
H	-4.32922	-3.97666	1.040342
C	-4.1259	3.723525	-0.00454
H	-4.00662	4.657471	-0.55548
H	-4.99231	3.187754	-0.39399
H	-4.32903	3.97697	1.040237

I5 (Charge: −1, Singlet)

C	2.523578	1.320011	0.123098
C	1.473652	-0.00036	-2.06115
C	2.523598	-1.31972	0.123571
O	3.3316	2.13132	0.30697
O	1.710884	-0.00073	-3.19515
O	3.331603	-2.13097	0.30777
N	-0.55796	-1.32046	-0.22255
C	-0.49527	-2.66002	-0.26631
C	-1.76658	-0.73858	-0.12029

C	-1.61063	-3.46504	-0.19995
H	0.494136	-3.0891	-0.35427
C	-2.93213	-1.49663	-0.05573
C	-1.76664	0.73848	-0.12034
C	-2.87465	-2.88236	-0.08647
H	-1.49301	-4.5412	-0.23639
H	-3.89507	-1.01038	0.021624
C	-2.93224	1.496438	-0.05575
N	-0.55807	1.320453	-0.22274
C	-2.87488	2.88218	-0.08657
H	-3.89513	1.010123	0.021726
C	-0.49549	2.660006	-0.2666
C	-1.61091	3.464952	-0.2002
H	-1.49338	4.541119	-0.23672
H	0.493872	3.089157	-0.35468
Re	1.186281	0.000079	-0.14658
O	-0.45918	0.00031	2.443406
C	0.753876	0.000183	2.086045
O	1.713349	0.000038	2.916687
C	-4.11165	-3.72685	-0.00104
H	-5.01445	-3.11553	-0.00611
H	-4.15747	-4.42555	-0.83972
H	-4.10094	-4.31905	0.917856
C	-4.11193	3.726571	-0.00103
H	-4.1577	4.425539	-0.83949
H	-5.01469	3.115213	-0.00638
H	-4.10135	4.318482	0.918053

Re(COOH) (Charge: 0, Singlet)

Re	1.172296	0.000052	-0.16046
C	2.53566	1.319837	-0.01112
C	1.335162	0.00008	-2.10225
C	2.535725	-1.31967	-0.01112
O	3.35679	2.126609	0.089274
O	1.468748	-3.1E-05	-3.24513
O	3.356898	-2.12639	0.089311
N	-0.58316	-1.32272	-0.17938

C	-0.51627	-2.66156	-0.20394
C	-1.79383	-0.74025	-0.09876
C	-1.63316	-3.46689	-0.14641
H	0.473966	-3.0917	-0.27186
C	-2.95744	-1.49711	-0.03936
C	-1.79386	0.740221	-0.09873
C	-2.8978	-2.88509	-0.05919
H	-1.51374	-4.54302	-0.16968
H	-3.92173	-1.01177	0.022831
C	-2.9575	1.497037	-0.03925
N	-0.58322	1.322741	-0.17937
C	-2.89791	2.885019	-0.05903
H	-3.92176	1.011649	0.022994
C	-0.51638	2.661585	-0.20389
C	-1.63331	3.466867	-0.14629
H	-1.51393	4.543011	-0.16952
H	0.473833	3.091774	-0.27183
C	0.830767	0.000068	2.01004
O	-0.25206	-0.00028	2.57788
O	1.944891	-0.00031	2.81162
C	-4.13549	3.728543	0.011503
H	-4.17909	4.412087	-0.83965
H	-5.03763	3.116741	0.014978
H	-4.12508	4.336931	0.919485
C	-4.13534	-3.72867	0.011298
H	-4.12513	-4.33673	0.919504
H	-5.03752	-3.11692	0.01433
H	-4.17868	-4.41252	-0.83962
H	1.632565	-0.00051	3.729212

I-4 (Charge: −1, Singlet)

Symbol	X	Y	Z
C	2.438675	1.404698	0.005094
C	2.156918	-0.60614	-1.73722
C	2.457568	-1.13137	0.844179
O	3.152077	2.316336	0.125182
O	2.728235	-0.93645	-2.69911

O	3.181195	-1.78195	1.483184
N	-0.35518	-1.42429	-0.03854
C	-0.2359	-2.77932	0.167742
C	-1.64538	-0.92374	-0.21187
C	-1.28474	-3.63451	0.23402
H	0.7791	-3.14159	0.278297
C	-2.77059	-1.80666	-0.14403
C	-1.70754	0.447711	-0.42987
C	-2.62393	-3.13892	0.076593
H	-1.10255	-4.68956	0.403921
H	-3.76392	-1.39257	-0.27609
C	-2.90939	1.203836	-0.60333
N	-0.46028	1.089889	-0.44024
C	-2.89359	2.546674	-0.80168
H	-3.85851	0.680011	-0.57135
C	-0.47803	2.45393	-0.67104
C	-1.6033	3.182921	-0.84355
H	-1.52137	4.251146	-1.01077
H	0.493454	2.930785	-0.69905
Re	1.248757	-0.07672	-0.18399
O	-1.17776	0.683658	2.853897
C	-0.526	1.628581	2.70994
O	0.112486	2.588952	2.61034
C	-3.78601	-4.08732	0.153276
H	-4.7354	-3.56743	0.009574
H	-3.70421	-4.86828	-0.60977
H	-3.81553	-4.59155	1.124743
C	-4.13925	3.365815	-0.98138
H	-4.13567	3.879221	-1.94878
H	-5.03532	2.743752	-0.9293
H	-4.21561	4.139518	-0.21006

TS2 (Charge: −1, Singlet)

C	2.422622	1.330049	0.305187
C	1.866443	-0.00033	-2.01643
C	2.422518	-1.33012	0.305543
O	3.162998	2.148259	0.677771

O	2.349093	-0.00049	-3.07899
O	3.162833	-2.14829	0.678339
N	-0.49492	-1.28375	-0.25583
C	-0.44112	-2.65302	-0.27099
C	-1.7432	-0.70963	-0.22534
C	-1.53919	-3.45272	-0.2293
H	0.552495	-3.07966	-0.31607
C	-2.9113	-1.51203	-0.18262
C	-1.74316	0.709651	-0.2254
C	-2.83966	-2.87639	-0.17738
H	-1.41137	-4.52906	-0.23947
H	-3.88078	-1.02967	-0.15342
C	-2.91121	1.512131	-0.18274
N	-0.49485	1.283691	-0.25597
C	-2.8395	2.876486	-0.17763
H	-3.88071	1.029824	-0.15348
C	-0.44097	2.652962	-0.27127
C	-1.53899	3.452728	-0.22964
H	-1.4111	4.529065	-0.23992
H	0.552666	3.079544	-0.31642
Re	1.182259	-6.5E-05	-0.25873
O	-0.81929	0.000189	2.550612
C	0.358153	0.000381	2.628295
O	1.383591	0.000671	3.21943
C	-4.05729	-3.75367	-0.13018
H	-4.97367	-3.16163	-0.0997
H	-4.09945	-4.4071	-1.00692
H	-4.03654	-4.39989	0.752591
C	-4.05707	3.75384	-0.13046
H	-4.09911	4.407365	-1.00713
H	-4.97349	3.161853	-0.10012
H	-4.03636	4.399959	0.752387

References

1. K. Kamogawa, Y. Shimoda, K. Miyata, K. Onda, Y. Yamazaki, Y. Tamaki and O. Ishitani, *Chem. Sci.*, 2021, **12**, 9682-9693.
2. E. Hasegawa, T. Seida, N. Chiba, T. Takahashi and H. Ikeda, *J. Org. Chem.*, 2005, **70**, 9632-9635.
3. X. Q. Zhu, M. T. Zhang, A. Yu, C. H. Wang and J. P. Cheng, *J. Am. Chem. Soc.*, 2008, **130**, 2501-2516.
4. K. Ohkubo, Y. Yamazaki, T. Nakashima, Y. Tamaki, K. Koike and O. Ishitani, *J. Catal.*, 2016, **343**, 278-289.
5. D. H. Gibson and X. Yin, *J. Am. Chem. Soc.*, 1998, **120**, 11200-11201.
6. M. D. Sampson, J. D. Froehlich, J. M. Smieja, E. E. Benson, I. D. Sharp and C. P. Kubiak, *Energy Environ. Science*, 2013, **6**.
7. Frisch, M. J.; Trucks, G. W.; Schlegel, H. B.; Scuseria, G. E.; Robb, M. A.; Cheeseman, J. R.; Scalmani, G.; Barone, V.; Petersson, G. A.; Nakatsuji, H.; Li, X.; Caricato, M.; Marenich, A. V.; Bloino, J.; Janesko, B. G.; Gomperts, R.; Mennucci, B.; Hratchian, H. P.; Ortiz, J. V.; Izmaylov, A. F.; Sonnenberg, J. L.; Williams, F.; Ding, F.; Lipparini, F.; Egidi, J.; Goings, B.; Peng, A.; Petrone, T.; Henderson, D.; Ranasinghe, V. G.; Zakrzewski, J.; Gao, N.; Rega, G.; Zheng, W.; Liang, M.; Hada, M.; Ehara, K.; Toyota, R.; Fukuda, J.; Hasegawa, M.; Ishida, T.; Nakajima, Y.; Honda, O.; Kitao, H.; Nakai, T.; Vreven, K.; Throssell, J. A.; Montgomery, A., Jr.; Peralta, J. E.; Ogliaro, F.; Bearpark, M. J.; Heyd, J. J.; Brothers, E. N.; Kudin, K. N.; Staroverov, V. N.; Keith, T. A.; Kobayashi, R.; Normand, J.; Raghavachari, K.; Rendell, A. P.; Burant, J. C.; Iyengar, S. S.; Tomasi, J.; Cossi, M.; Millam, J. M.; Klene, M.; Adamo, C.; Cammi, R.; Ochterski, J. W.; Martin, R. L.; Morokuma, K.; Farkas, O.; Foresman, J. B.; Fox, D. J. *Gaussian 16*, revision C.01; Gaussian, Inc.: Wallingford, CT, 2016.
8. A. W. Ehlers, M. Böhme, S. Dapprich, A. Gobbi, A. Höllwarth, V. Jonas, K. F. Köhler, R. Stegmann, A. Veldkamp and G. Frenking, *Chem. Phys. Lett.*, 1993, **208**, 111-114.
9. J. A. Keith and E. A. Carter, *J. Chem. Theory Comput.*, 2012, **8**, 3187-3206.
10. T. Nakajima, Y. Tamaki, K. Ueno, E. Kato, T. Nishikawa, K. Ohkubo, Y. Yamazaki, T. Morimoto and O. Ishitani, *J. Am. Chem. Soc.*, 2016, **138**, 13818-13821.
11. T. Morimoto, T. Nakajima, S. Sawa, R. Nakanishi, D. Imori and O. Ishitani, *J. Am. Chem. Soc.*, 2013, **135**, 16825-16828.
12. H. Koizumi, H. Chiba, A. Sugihara, M. Iwamura, K. Nozaki and O. Ishitani, *Chem. Sci.*, 2019, **10**, 3080-3088.
